# Social-ecological system analysis of an invertebrate gleaning fishery on the island of Unguja, Zanzibar

**DOI:** 10.1007/s13280-022-01769-1

**Published:** 2022-08-09

**Authors:** Johann Stiepani, Narriman Jiddawi, Lina Mtwana Nordlund

**Affiliations:** 1 Natural Resources and Sustainable Development, NRHU Department of Earth Sciences, Uppsala University, Campus Gotland, Visby, Sweden; 2 Institute of Marine Sciences, University of Dar Es Salaam, Dar Es Salaam, Tanzania

**Keywords:** Invertebrate gleaning, Small-scale fisheries, Social-ecological systems, Western Indian Ocean

## Abstract

Invertebrate gleaning is a small-scale fishery that commonly occurs in the intertidal zone across the tropical Indo-Pacific. In this study, we investigated and analyzed several components of this fishery on the island of Unguja, Zanzibar by employing the social-ecological systems framework from Ostrom 2009. In doing so, we conducted ecological surveys, catch assessments, interviews with gleaners, household surveys, focus group interviews and analyzed the governance structure. This social-ecological systems analysis showed that gleaning is important for food security, local culture and livelihood. Yet, the multiple approaches in our study revealed that the local intertidal zone is degrading and that the gleaned catch is changing. Local narratives indicate that economically important bivalves (*Modiolus* spp.) and gastropods (*Strombus* spp.) are in decline, which was paralleled with low abundances of both genera within the ecological survey of the intertidal and catch landing assessment. We recommend that invertebrate gleaning, a fishery mainly comprised of women should be included in fisheries management.

**Supplementary Information:**

The online version contains supplementary material available at 10.1007/s13280-022-01769-1.

## Introduction

The coastal zone is one of the most productive environments and is supporting a wide range of ecosystem services contributing to human wellbeing (Barbier et al. 2011; Lau et al. 2019). Many millions of people along the coasts are directly supported by this complex social-ecological system (SES), for example through small-scale fisheries (SSF) (Teh and Sumaila 2013; Béné et al. 2015). SSF offer food security and livelihoods to many coastal communities within the Indo-Pacific (Teh and Sumaila 2013; Kittinger et al. 2013; Unsworth et al. 2019). Yet, a common reality within the tropical Indo-Pacific is that unsustainable fishing practices degrade these SES (Cinner 2009, 2011; Gough et al. 2020).

Within the tropical Indo-Pacific, one common but less studied SSF is invertebrate gleaning, i.e. fishing by walking in the intertidal zone (Harper et al. 2013, 2020; Nordlund et al. 2018; Unsworth et al. 2019). Invertebrate gleaning requires little to no gear, is commonly conducted by women and often occurs near the gleaner’s place of residence (Crawford et al. 2010; Nordlund et al. 2010; de la Torre-Castro et al. 2017; Furkon et al. 2020; Alati et al. 2020). Gleaning is a multi-species SSF where anywhere from one to hundreds of different species are targeted and species from the phylum Mollusca compose the majority of catch in these SSF (Kyle et al. 1997; Crawford et al. 2010; Nordlund et al. 2010; Nordlund and Gullström 2013; Fröcklin et al. 2014; Furkon et al. 2020). Gleaning can have negative impacts on local invertebrate stocks and damage coastal ecosystems, but more studies are needed to better understand these environmental consequences (Nordlund and Gullström 2013; Tania et al. 2014; Alati et al. 2020). Even though gleaning is very common, these female-dominated SSFs are often overlooked in fishery management (Unsworth and Cullen 2010; Nordlund et al. 2010, 2014; Kleiber et al. 2014), yet the inclusion of gleaning into local management and monitoring could be one way to better understand the dynamics of these SES.

SES thinking provides a holistic understanding of the intertwinedness of the various social and ecological components that constitute these coupled systems (Ostrom 2009; Folke et al. 2010, 2016; Cullen-Unsworth et al. 2014). Results from a social-ecological analysis can diagnose unsustainable social-ecological interactions and outcomes at the local level in the context of environmental governance (Ostrom 2009; Partelow et al. 2019). Local ecological knowledge (LEK) is especially important when long term monitoring of the common pool resource is missing with the example of data-poor fisheries (Johannes et al. 2000; Rehren et al. 2020; Alati et al. 2020). LEK is defined as knowledge held by a specific group of people about their local ecosystems (Olsson and Folke 2001; Berkström et al. 2019). Drawing from different knowledge types and data sources during field investigation i.e. social and ecological surveys can be corroborated to understand the status of a common pool resource and the outcomes of social-ecological interactions (Epstein et al. 2013; Berkström et al. 2019; Rehren et al. 2020).

Considering research on invertebrate gleaning, prior investigations on invertebrate gleaning fisheries have analyzed one or several components of these SES. Previous research has investigated, for example, impacts of seasonality on catch composition (Kyle et al. 1997; Grantham et al. 2021), LEK of invertebrate gleaners (Alati et al. 2020), time series of gleaned catch (Fröcklin et al. 2014), and linkages of gleaning to human well-being (Cullen-Unsworth et al. 2014; Grantham et al. 2020). While effort has been placed on different aspects of invertebrate gleaning, few studies have considered investigating invertebrate gleaning as a SES (Wosu 2017; Furkon et al. 2020).

This study uses an interdisciplinary research approach aiming to more holistically understand the nuances of invertebrate gleaning as a SES. The SES was investigated using several methods, namely ecological surveys, catch assessments, interviews with gleaners, household surveys, focus group interviews and analyzing the governance structure in the rural village, Unguja Ukuu, on the island of Unguja, Zanzibar in Tanzania. In this gleaning fishery, we investigated the resource system (the intertidal zone where invertebrate gleaning occurs), the resource users (invertebrate gleaners), the resource unit (invertebrates), and the governance system (the local governance and management concerns and regulations of invertebrate gleaning and co-occurring SSF). We then discuss the value of using multiple approaches for a deeper understanding of the system to be able to make policy recommendations to enhance the sustainability of the invertebrate gleaning fishery.

## Materials and methods

### Theoretical framework: Social-ecological systems framework

**Fig. 1:**
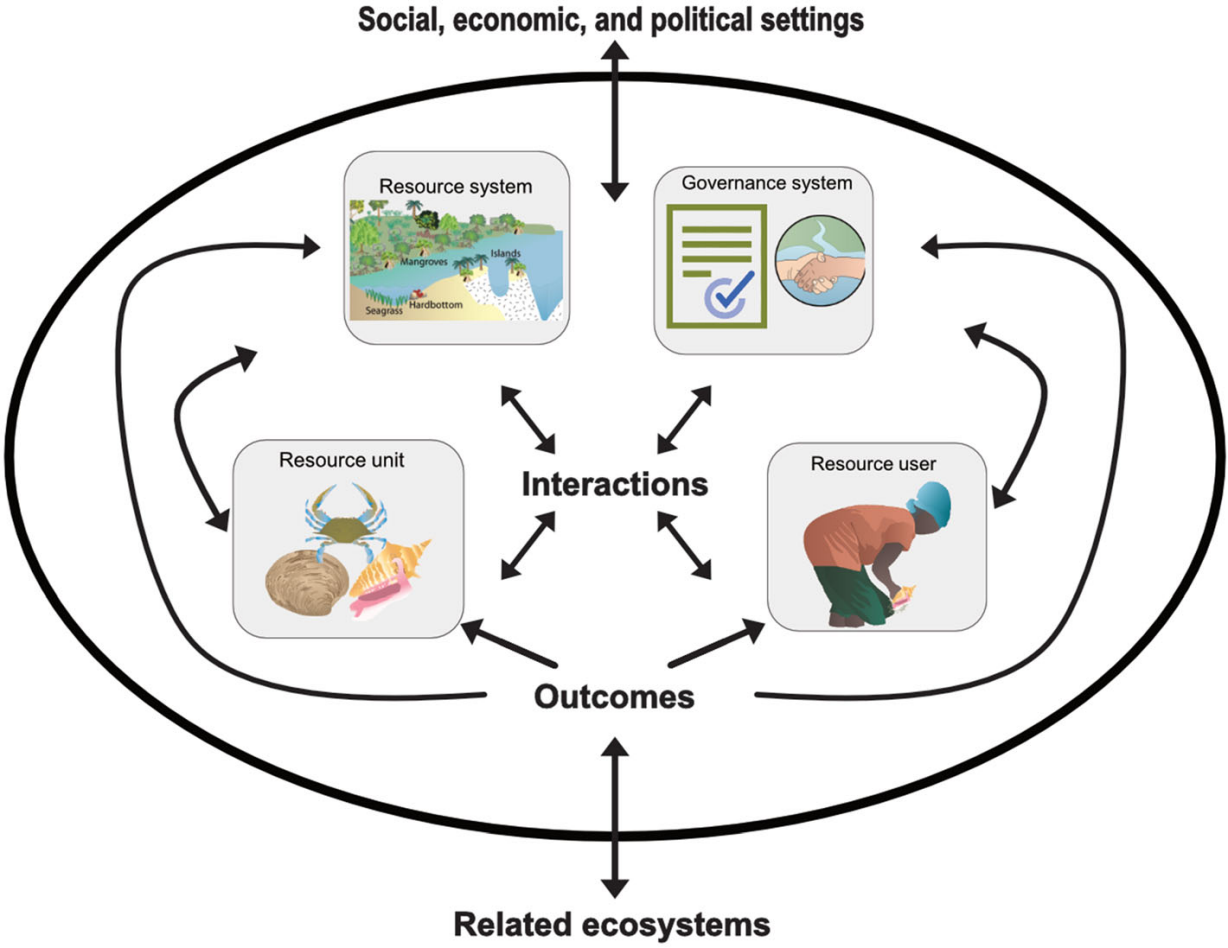
Gleaning depicted as a social-ecological system using the social-ecological system framework amended from Ostrom (2009)

The SES framework is a generalized framework that can be used to investigate the sustainable usage of a common pool resource (Ostrom 2009). The SES framework separates SES into four different components which are named the resource system, the resource unit, the resource user, and the governance system (Fig. 1). The SES framework can be used as a diagnostic tool for assessing negative and positive interactions and outcomes among the resource system, resource unit, resource user, and governance system (Ostrom 2009). Resource users and various actors within the SES are influenced by the current and past social, economic, and political setting and by related ecosystems which are important to understanding the context of a SES (Ostrom 2009). The SES framework can guide an interdisciplinary analysis of any SES (Ostrom 2009). Using a common framework like SES framework provides a common language to make a comparison of the different components of a SES, their interactions, and outcomes. The ability to compare different SES can provide insights into collective actions that solve common pool resource dilemmas or to understand why other SES are unsustainable.

### Description of the study site

**Fig. 2:**
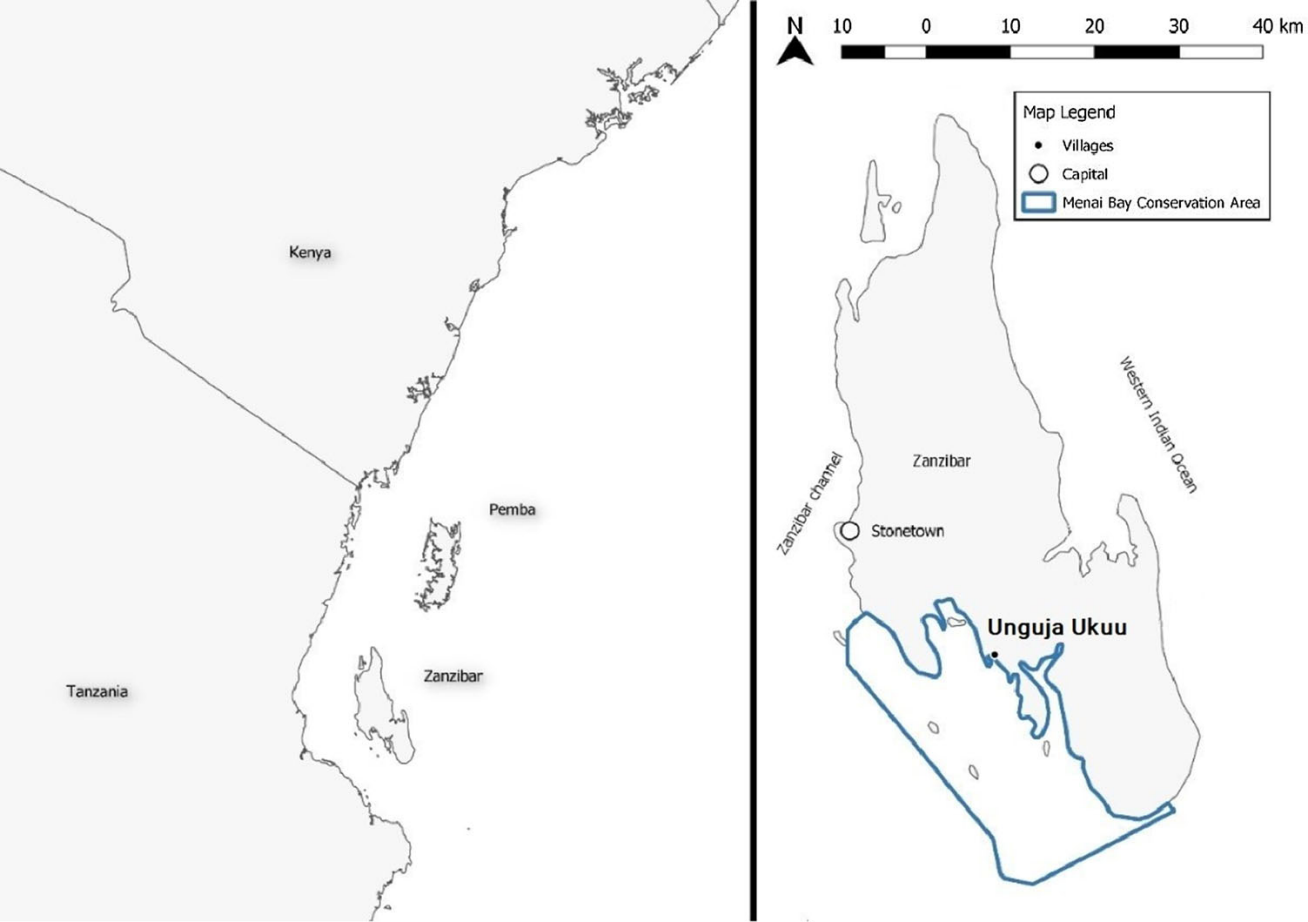
Map of study site Unguja Ukuu, Zanzibar, Tanzania

This case study was carried out on the island of Unguja or commonly known as and referred to in this paper as Zanzibar. Zanzibar is a semi-autonomous region of Tanzania located approximately 50 km east of the mainland of Africa. The total land area of Zanzibar is 2,461km^2^ and is flanked by the tropical waters of the Western Indian Ocean (WIO) to the east and the Zanzibar channel to the west. Fieldwork was conducted in a rural fishing village Unguja Ukuu from June to August 2019 (Fig. 2). The coastal waters of Unguja Ukuu lay within the Menai Bay Conservation Area (MBCA). MBCA is the largest marine conservation area (467 km^2^) in Zanzibar and hosts seagrass meadows, mangrove forests, and coral reefs within the coastal zone (Khamis et al. 2017). Zanzibar has a tropical climate with two distinct seasons driven by the monsoon; the rainy seasons from October to December (Kaskazi) and from March to May (Kuzi) (Khamis et al. 2017). During the time of fieldwork, there was a dry period with a few rainy days. The tides are semi-diurnal with a maximum tidal range of just over four meters during spring high tide (Hedberg et al. 2018).

Rural communities in Zanzibar, like Unguja Ukuu, are heavily reliant on marine resources for their livelihood and food security (Jiddawi and Öhman 2002; Khamis et al. 2017). The vast majority of SSF occurs within nearshore coastal areas placing continual pressure on coastal and intertidal ecosystems (Jiddawi and Öhman 2002; Rehren et al. 2020). Gleaning fisheries similiar to the one found at Unguja Ukuu are common within the intertidal zone around the island of Unguja in Zanzibar (de la Torre-Castro and Rönnbäck 2004; Crawford et al. 2010; Nordlund et al. 2010; Fröcklin et al. 2014; de la Torre-Castro et al. 2017; Rehren et al. 2020).

### Ecological assessment of the intertidal

To assess the intertidal zone in more detail 33 individual 50 m transects, in sets of three, were laid out in the intertidal zone during spring low tide perpendicular to the shore. This was done to assess the availability of invertebrates for the gleaners to collect. Two different habitat types were selected for the survey; vegetated areas dominated by seagrass and unvegetated areas (areas with less than 10% vegetation). Eighteen transects were conducted in the vegetated areas and fifteen transects were conducted in unvegetated areas. For each transect, first the abundance of larger invertebrates (> 5 cm) was recorded along the transect line in a belt transect manner within one meter on each side of the transect line (total of 100 m^2^ per transect). After the belt transect, 0.25 m^2^ quadrats were laid along the transect every 5 m to record information about the habitat. In each quadrat visual observations of seagrass coverage (%), seagrass species composition (%), macroalgae coverage (%), and substrate type were recorded. In four of the 0.25 m^2^ quadrats (at 0 m, 15 m, 35 m, 50 m) along each transect the abundance of small invertebrates > 1 cm up to 5 cm was recorded. Further details on the ecological assessment can be found in the (Supplementary information 2). Species were identified to the lowest taxonomical level possible in the field with a non-invasive visual approach.

### Data analyses of the ecological assessment

All analyses were calculated using R 4.0. Seagrass species frequency was calculated as number of quadrats where the species occurred divided by the total number of quadrats multiplied by 100. To make a comparison of the invertebrate abundances collected within the large and small methods; abundance data was transformed into invertebrate density at the spatial resolution of 1 m^2^. Thus, the large invertebrate abundances which were collected with 100 m^2^ transects was divided by 100 and the small invertebrate abundances which were collected with 0.25 m^2^ quadrats was multiplied by four. After the invertebrate abundance was adjusted to density at the spatial resolution of 1 m^2^, the following statistical tests were performed. To compare the similarities of invertebrate community composition between vegetated and unvegetated transects a 1-way ANOSIM was conducted based on Bray–Curtis Similarity Index with 9,999 Monte Carlo permutations after a 4th root transformation of the invertebrate density data. A SIMPER test was performed to reveal each species contribution to dissimilarities of invertebrate densities between vegetated and unvegetated transects. To compare individual species densities between vegetated and unvegetated transects, the non-parametric Mann–Whitney U-test was performed, since all invertebrate densities were found to vary non-parametrically.

### Gleaning landing survey: Interview and catch assessment

In all social components of this study, verbal consent was given prior to the interview. Each interviewee understood the purpose of the study, the potential output, and how their response could be used. Each interviewee understood that their response would be anonymous and that they could exit or withdraw from the interview at any point. If consent was not granted from the interviewee, the interview was not conducted.

To solicit information on the invertebrate gleaner’s preference when gleaning and their landed catch, a landing survey was conducted with gleaners on the beach after they returned from gleaning. In total, 29 invertebrate gleaner landing surveys were conducted on the beach. The interviews were held during spring low tide during daytime as it is the most common time for gleaning. Gleaner landing surveys consisted of two components. The first component was a semi-structured interview conducted with the aid of a translator and the second component was an assessment of each gleaner’s catch. The interview forms can be found in the Supplementary information 1, Table S1. The invertebrates were identified to species level (or lowest taxonomical level possible with a non-invasive in-field visual approach) and abundances of each species were recorded. Each landing survey lasted 15–45 min.

### Gleaner household survey

Household surveys, in form of semi-structured interviews, were conducted with gleaners from the village Unguja Ukuu to solicit additional information about the local invertebrate gleaning fishery in the context of food security, livelihood, and cultural value. The interview form can be found in the Supplementary information 1, Table S2. With the assistance of a key fishery informant, we were introduced to households with gleaners within the village of Unguja Ukuu who were available at the time of the study for a household survey. The gleaners that partook in the household survey were not necessarily the same individuals that partook in the gleaner landing survey. With the assistance of a translator, a semi-structured interview took place at the household of each gleaner. The household interview allowed for flexibility in response and allowed for follow up questions. Pictures were taken when permitted to show usages of gleaned catch at the household. Each household interview lasted 30–60 min.

### Focus session with invertebrate gleaners and fisherfolk of Unguja Ukuu

To solicit information on local SSF and general coastal management concerns; a focus session was held with local community members of Unguja Ukuu. Two translators assisted in the facilitation of the focus session. A semi-structured protocol was used during the 150-min focus session. This semi-structured protocol can be found in the Supplementary information 1, Table S3.

### Governance system analysis

The Fisheries Act of 2010, the current legislation for all fisheries within the Exclusive Economic Zone of Zanzibar, was thoroughly reviewed. The review focused on identifying how gleaning is regulated and the legality of commonly co-occurring SSF at the study site of Unguja Ukuu. Additionally, the Zanzibar Fisheries Frame Survey of 2016 (MANLF 2017) and a draft of Zanzibar Fisheries Survey 2020 (DFD 2020) were reviewed to assess if gleaning is considered in government assessments and long term monitoring of SSF across the island of Unguja, Zanzibar.

## Results

### Overview of invertebrate gleaning as a social-ecological system

**Fig. 3:**
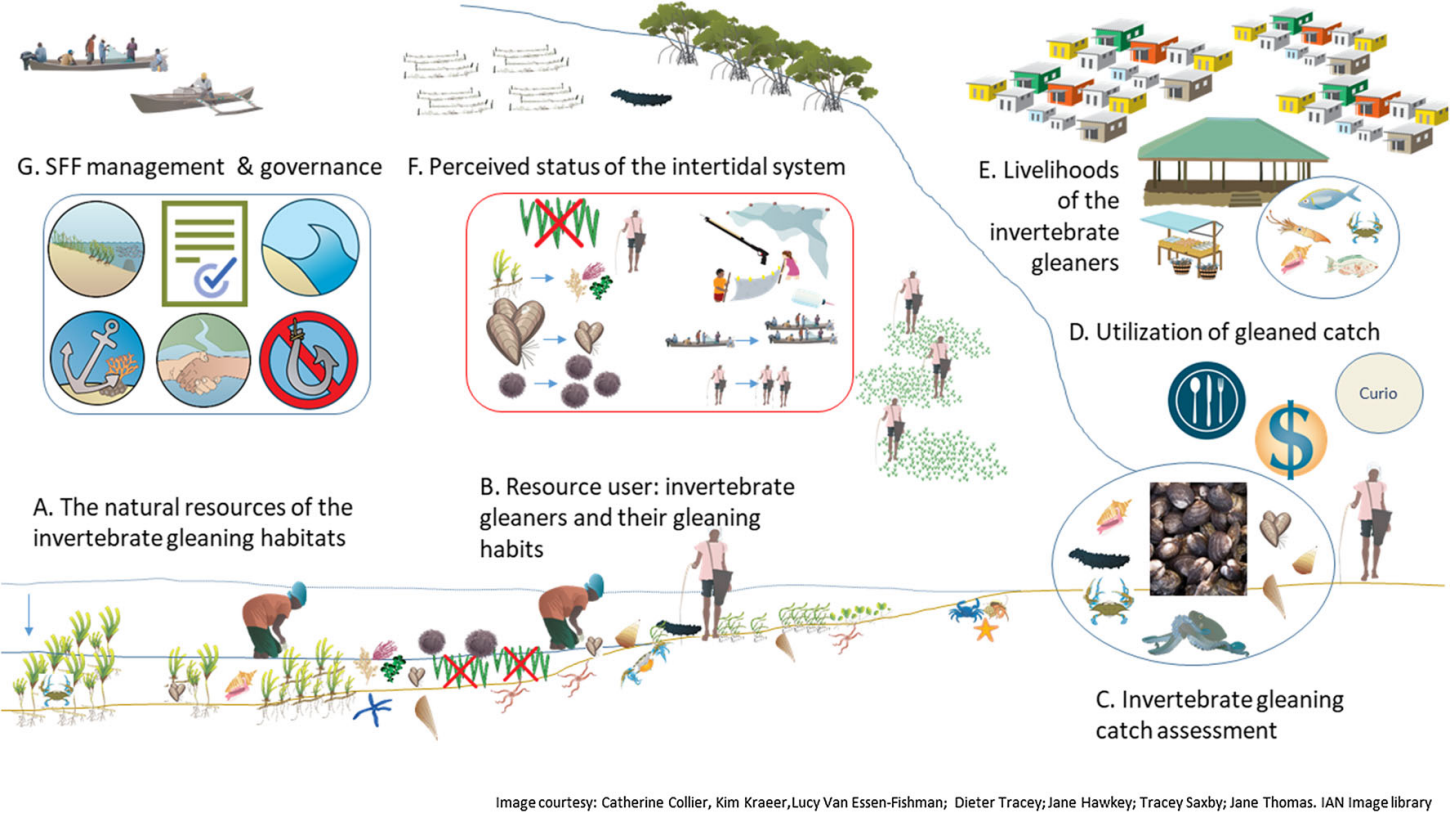
Schematic illustration of an intertidal zone and a local village in Zanzibar, Tanzania. Visualized are the different elements A-G of invertebrate gleaning as a social-ecological system. These elements include: **A** Natural system resources of the invertebrate gleaning habitats, with seagrass, algae and typical fauna found in this system, and fishing boats and seaweed farms, **B** Resource user: invertebrate gleaners and their gleaning habits, **C** Invertebrate gleaning catch assessment, the oval represents typical species landed, **D** Utilization of gleaned catch, illustrated by a plate, dollar sign and curio, **E** Livelihoods of the invertebrate gleaners, illustrated by houses and markets **F** Perceived status of the intertidal system, illustrated an increasing amount of gleaners and fishing boat, more urchins, smaller bivalves, and less seagrass and **G** SSF management and governance, illustrated by symbols related to governance and management

The four components of the SES framework (Fig. 1) were investigated as seven different elements (Fig. 3) using five different research methods. The elements are (A) the intertidal system with details about the habitats and associated species, (B) the resource user and their gleaning habits, (C) gleaning landing catch assessments, (D) the utilization of the catch, (E) livelihoods of the gleaners, (F) the perceived status of and the threats to the intertidal system and (G) analysis of governance and management. To gain a better understanding of the present state of the invertebrate gleaning fishery we synthesize the results of the seven elements of the SES to highlight several key social-ecological interactions and outcomes of the resource system, resource unit, resource user, and governance system.

### The natural resources of the invertebrate gleaning habitats

Visual observations of the intertidal zone in Unguja Ukuu, reveal that the intertidal zone is dominated by sparse to dense seagrass with sandy, muddy and rocky patches, and parts of the coastline are lined with mangroves. The ecological survey with 33 transects placed in the intertidal seagrass habitat (vegetated) and unvegetated areas (> 10% vegetation cover) showed that the most commonly observed substrate within the quadrats was sand (82.69%) followed by sand covering limestone rock (16.42%), then mud covering rock (0.89%). Macroalgae were evenly distributed between vegetated and unvegetated transects with an overall macroalgae coverage of 4.62% (SD ± 4.12). The seagrass system where gleaning was most frequently observed is species-rich with eight seagrass species recorded within vegetated transects (ordered from highest species frequency) including; *Thalassia hemprichii* (94.44%) *Halophila ovalis* (44.44%), *Halodule uninervis* (33.33%), *Cymodocea rotundata* (22.22%), *Cymodocea serrulata* (22.22%), *Enhalus acoroides* (16.67%), *Thalassodendron ciliatum* (16.67%), and *Syringodium isoetifolium* (3.03%).

A total abundance of 2170 invertebrates was recorded within the ecological assessment. Collectively, a species richness of 29 invertebrates was recorded from 23 different Families and Superfamilies (Supplementary information 1, Table S4, S5). The density composition of the invertebrate community did not vary significantly between vegetated and unvegetated transects (1-way ANOSIM, *R* = − 0.019, *p* = 0.6). The SIMPER test revealed *Clibanarius* spp. (41.35%), *Cypraea annulus* (8.35%), *Edwardsianthus* spp*.* (5.74%), and *Trochus* spp*.* (5.44%) contributed the highest to the dissimilarity to the density of the invertebrate community between vegetated and unvegetated transects (Supplementary information 1, Table S4). The SIMPER test found all other species densities contributed less than 5% to the average dissimilarity in invertebrate community composition between seagrass and unvegetated transects (Supplementary information 1, Table S4). At the species level the density of only one species, the sea urchin, *Echinometra mathaei* varied significantly between vegetated and unvegetated transects (Mann Whitney *U* test, *U* = 76.5, *p* < 0.029) with a higher density within vegetated transects (Supplementary information 1, Table S4).

### Resource user: Invertebrate gleaners and their gleaning habits

Three different protocols with different foci were used to learn more about the gleaners and their gleaning practices. Twenty-nine gleaners were interviewed during gleaning landing surveys, 36 gleaners during household surveys, and 14 gleaners during the community focus session. Additionally, four fishermen attended the community focus session. In total, invertebrate gleaners were interviewed 79 times, all adults (≥ 18 years). Gleaners were interviewed only once in each of the different interview surveys but not all gleaners were available for each the landing survey, household survey, or the focus session.

Of the 36 gleaners interviewed during the household survey 72% (*n* = 26) had a secondary education, 19% (*n* = 7) had primary education, and 9% (*n* = 3) had no education. The household survey found a mean age of 38 (SD ± 11.5) years. When interviewed the average career length for an individual participating in gleaning was 17.5 years (SD ± 9), indicating on average participants in our study have many years of experience in this fishery. The oldest gleaner interviewed during the household survey was a 75 years old woman and the youngest invertebrate gleaner found at the study site was 12 years old (but was not interviewed as we only interviewed people 18 years of age or older).

**Fig. 4:**
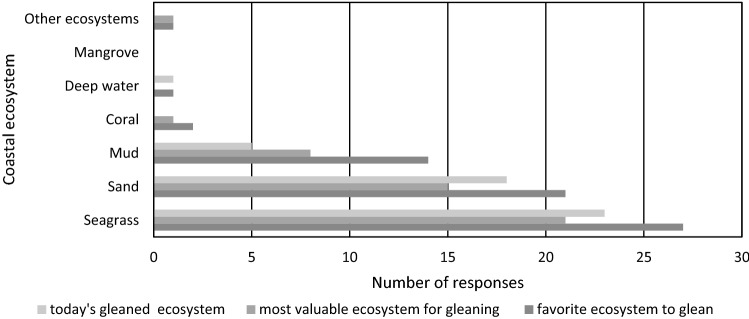
Preferred ecosystems for gleaning; The colors of the bars represent: today’s gleaned ecosystem—includes all ecosystems where they had been gleaning during the day of the gleaning landing survey; most valuable ecosystem for gleaning—shows the respondents’ preferences for finding the most valuable catch in monetary terms; and favorite ecosystem to glean—shows their favorite ecosystem to glean in general

During the 29 invertebrate gleaning landing surveys, the vast majority of gleaners reported that seagrass was their favorite ecosystem to glean in (*n* = 27) and when asked in which ecosystem they get the most valuable catch (in monetary terms) (*n* = 21). Sand was the second most selected option for favorite ecosystem to glean in and where they get their most valuable catch (Fig. 4). When asked where they had been gleaning on the day of the survey 23 gleaners had been gleaning in the seagrass, 18 in sand and 5 in mud, i.e. several gleaners had gleaned in multiple ecosystems (Fig. 4). No gleaners reported gleaning for invertebrates in mangrove forests or coral reefs on the day of the survey since both are ecosystems are not favorable or locations where valuable catch can be collected (Fig. 4).

All the 14 invertebrate gleaners at the focus session reported that the seagrass and sand are the most important ecosystems for invertebrate gleaning in comparison to mangroves and coral reefs. Several of the gleaners reported, a preference for gleaning within seagrass with mud substrate while other gleaners prefer seagrass ecosystems with sand substrate. Several gleaners preferred to glean during spring low tide when seagrass meadows are fully exposed while other gleaners favored water at knee height due to the availability of their preferred species to be caught.

During the 29 invertebrate gleaning landing surveys, the gleaners reported on average their gleaning effort to be 16.9 days (SD ± 6.4) per month. Gleaners reported an average of 4 h (SD ± 1.1) of gleaning on a typical day (two gleaners did not respond to this question since they did not remember). On the day of the survey, the average time for gleaning was 2.9 h (SD ± 0.8). Furthermore, on the day of the survey 24 gleaners (82%) did report spending time gleaning in the seagrass while five gleaners (18%) reported not gleaning within the seagrass. On the day of the survey, gleaners reported spending 1.9 h (SD ± 0.8) within the seagrass ecosystem or an estimated 65% of their time.

### Invertebrate gleaning catch assessment

During the 29 invertebrate gleaning catch assessments, an invertebrate species richness of 44 was recorded; comprising 31 different Families and Superfamilies (Supplementary information 1, Table S6). The total abundance of gleaned invertebrates was 2,116 individuals across 29 landings. The most abundant species was the cockle *Anadara antiquata* with 1,067 individuals counted, representing over 50% of the total abundance in this study (Supplementary information 1, Table S6). Other commonly recorded invertebrates in the catch assessment were *Chicoreus ramosus* (7.23%), *Pinctada margaritifera* (6.95%), *Cardiidae* spp*.* (5.39%), *Trochidae* spp*.* (4.77%), *Portunus pelagicus* (5.34%), and *Isognomon isognomum* (4.40%) but composed proportionally smaller compositions of the total catch (Supplementary information 1, Table S6). The remaining 37 species were recorded in smaller abundances and included three finfish species and one octopus species (Supplementary information 1, Table S6).

**Fig. 5:**
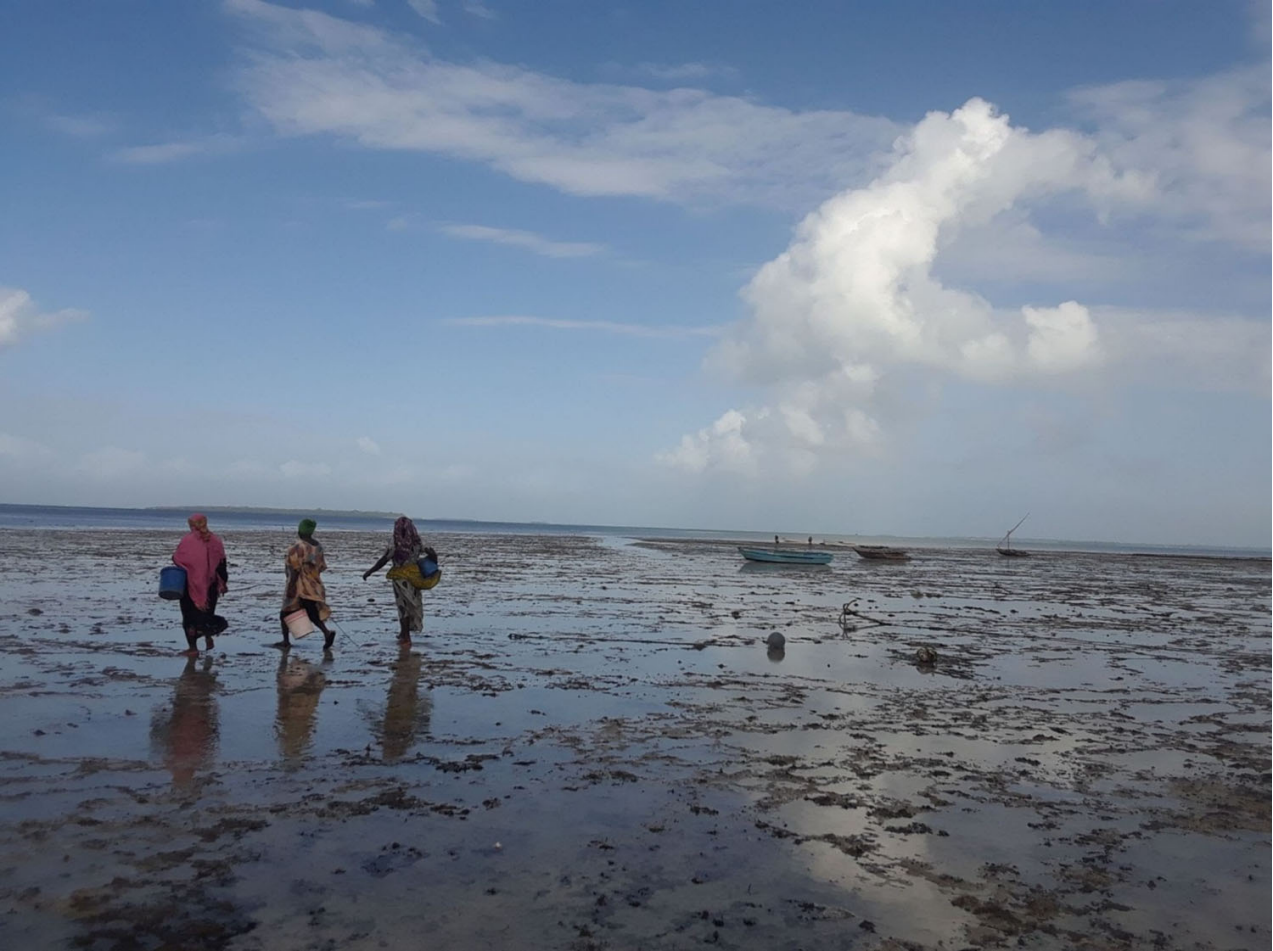
The image shows three women going out into the seagrass dominated intertidal zone to glean for invertebrates in the early morning during low tide in Zanzibar, Tanzania. These women are barefooted, carrying metal rods, and buckets to assist with collecting invertebrates. (Photograph: Johann Stiepani, 2019)

The average weight of a catch was 3.20 kg (SD ± 1.67) and the average catch per unit effort was 1.17 kg/h (SD ± 0.73). In comparison to previous catches, 27 out of 29 gleaners said their day's catch was smaller, while two gleaners reported their catch to be larger than usual. Of the 29 gleaners interviewed, 25 gleaners reported using tools while four gleaners used only their hands. The most common tools used while gleaning was metal rod (*n* = 21), followed by wooden stick (*n* = 2), and rocks (*n* = 3) (Fig. 5).

### Utilization of gleaned catch

During the 36 household surveys, invertebrate gleaners reported several reasons for gleaning (interviewees were able to select more than one response). The most frequent response was for additional income (*n* = 25), followed by for food (*n* = 14), as their only source of livelihood (*n* = 12), nothing else to do (*n* = 7), for social activities with their peers (*n* = 5), to sell as curio (*n* = 4), to add variety to their diet (*n* = 3), and for fishing bait (*n* = 3).

**Fig. 6:**
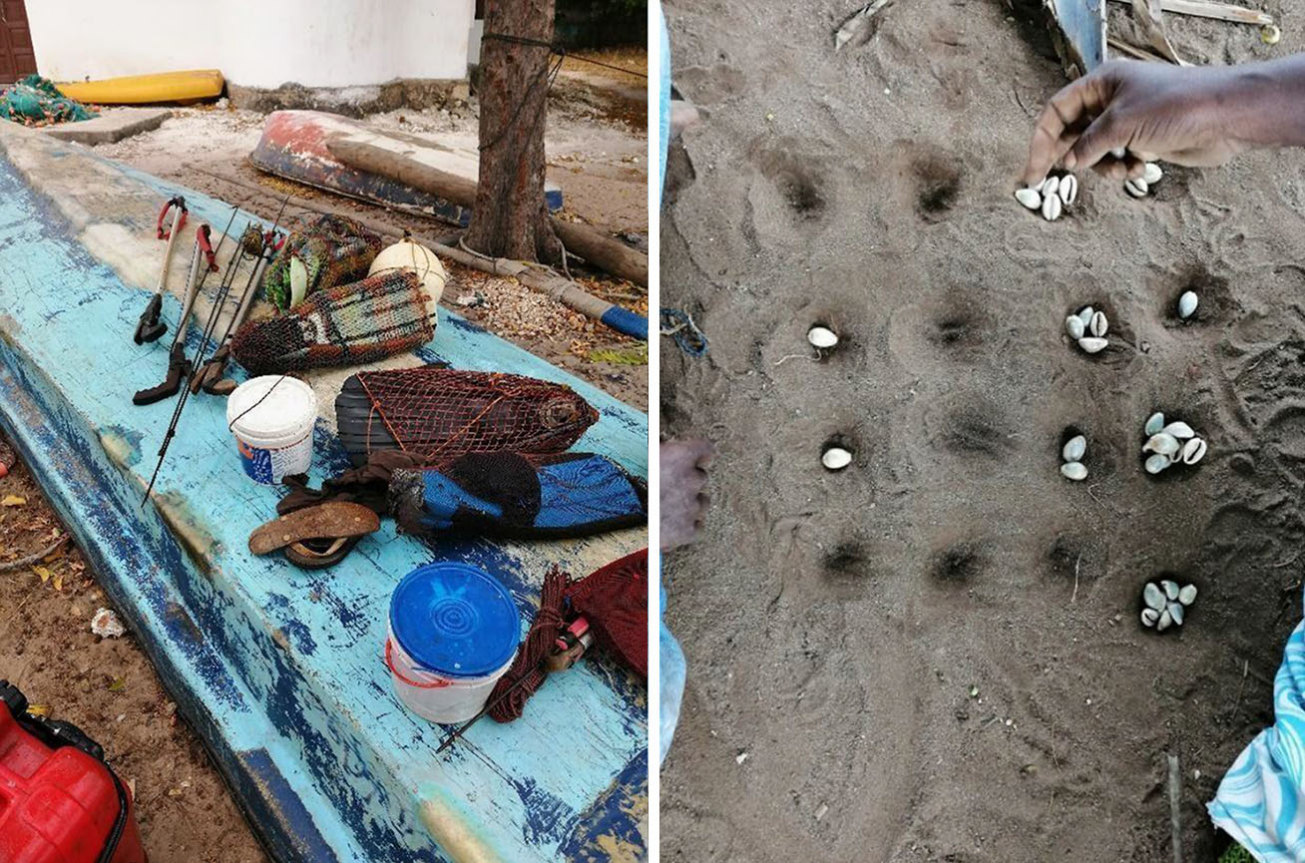
The image on the left shows illegal spearfishing gear at Unguja Ukuu, Zanzibar. The image on the right shows cultural usages of the gleaned invertebrates ring cowrie (*Monetaria annulus*) in traditional Bao game. This usage of the shell *M. annulus* was observed during household surveys. (Photograph: Johann Stiepani, 2019)

Gleaners reported utilizing their additional income from gleaning for a variety of purposes. The most common answer was for buying other food (*n* = 24) including rice, vegetables, and protein. The second most common answer was to buy clothing, dresses, or material for tailoring Kanga dresses (*n* = 13). Furthermore, income from gleaning was used for schooling their children (*n* = 9) and health care for their sick children (*n* = 2). Commonly, additional income was invested into the Saving and Credit Co-operative (SACCOS), a micro-financial institution with the goal of starting new businesses (*n* = 7). Furthermore, gleaners used their income to buy household items; for instance soap (*n* = 5) and furniture (*n* = 1). Income from gleaning was found to support cultural events including weddings (*n* = 2) or funerals (*n* = 1). During the household surveys, cultural usages of gleaned species were observed. In several households, the ring cowrie (*Monetaria annulus*) was used as game pieces in the traditional game Bao and was not sold for income (Fig. 6). When considering the contribution of invertebrate gleaning to the household’s food security, 58% (*n* = 21) stated that gleaning was important to their household’s food security, while 42% (*n* = 15) stated it was not so important since the main food source derived from other sources rather than from gleaning.

### Livelihoods of the invertebrate gleaners

During the 36 household surveys, two-thirds of the invertebrate gleaners (*n* = 24) reported no alternative livelihood to gleaning, while one-third of the gleaners (*n* = 12) reported having various alternative livelihoods to gleaning. Alternative livelihoods to gleaning consisted of selling bread, chapatti, or local cuisine (*n* = 6), tailoring dresses (*n* = 2), making makuti which is thatch roofing material (*n* = 2), running a small business (*n* = 1), and selling vegetables (*n* = 1). During the community focus session discussions, the 14 gleaners reported that they were able to sell their gleaned catch at the local fish auction of Unguja Ukuu, and smaller markets within the village, as well as along the roadside to Stonetown. During the community focus session, participants reported that an attempt for alternative livelihood in the form of a community-based sea cucumber farm had been implemented but it was not very successful. The focus session participants reported there was a lack of the community sustaining the effort of keeping the farm after the project initiation. Gleaners reported that poaching often occurred within the sea cucumber farm due to the need for food and income.

### Perceived status of the intertidal system

All 18 participants of the focus session reported that they have perceived a decrease in seagrass distribution and an increase in algae coverage within the intertidal zone. Participants reported that they had noted a change in seagrass species composition within the intertidal seagrass ecosystem. In the past, *Cymodocea rotunda* and *C. serrulata* were more commonly observed by the gleaners and fishermen. Their perception is that in recent times, the intertidal seagrass species composition has become more dominated by one species, *Thalassia hemprichii*. During the focus session, gleaners reported that several economically important invertebrate species have declined, including certain bivalves (*Modiolus* spp*.*) and gastropods (*Strombus* spp*.*) that have become extremely rare within the intertidal of Unguja Ukuu. The gleaners also reported an increase in sea urchin abundances (*Echinometra mathaei*).

During the 36 household surveys, 94% (*n* = 34) of the gleaners reported an overall decrease in invertebrate abundances in Unguja Ukuu. One gleaner reported that abundance remained the same while one other gleaner reported that there was an increase in invertebrate abundances. All gleaners in the household survey stated that glean catch composition varies between monsoon seasons. During the household survey, the gleaners were asked about threats to the provision of invertebrates within the intertidal and were allowed one or more responses. The most common response was that gleaners did not know what is threatening invertebrates within the intertidal zone (*n* = 16), followed by ecological change within the seagrass ecosystem (*n* = 10), increased wastewater (*n* = 10), climate change (*n* = 8), increases of gleaners at Unguja Ukuu (*n* = 7), industry and tourism (*n* = 3), and plastics and other waste (*n* = 1).

### SSF management and governance at Unguja Ukuu

During the 36 household surveys, 35 of the 36 invertebrate gleaners interviewed stated that they did not know what could be done to conserve invertebrates within the intertidal. While one gleaner stated that smaller invertebrates should not be taken from the intertidal until becoming larger in size.

The 14 gleaners and 4 fishermen who participated in the community focus session perceived that gleaning is not considered in SSF management. They also said that there is a lack of knowledge on coastal management among the group of participants in the focus session. Participants were interested in having trainings on coastal resource management but did not have the financial support to host a training on coastal resource management. Previously, a “no-take zone” was established to support the local gleaning fishery by protecting the spawning grounds for invertebrate species. Participants reported that the “no-take zone” failed due to a lack of community effort. Participants in the community focus group mentioned that poaching within the “no-take zone” commonly occurred. Illegal encroachment into the “no-take zone” was reported due to the need for livelihood and food.

A review of the Fisheries Act of 2010, shows that fishing using poison, illegal mesh sizes in fishing nets, and spearfishing are illegal fishing practices. According to the focus group participants, these fishing methods are used within the co-occurring fin fisheries at the study site. In addition, the review of the Fisheries Act of 2010, found that invertebrate gleaning is not explicitly defined as a SSF. After reviewing the Zanzibar Fisheries Frame Survey of 2016 and the draft Zanzibar Fisheries Frame Survey of 2020 (MANLF 2017; DFD 2020) it shows that gleaning is not included as a monitored fishery of Zanzibar. Although, within the Zanzibar Fisheries Frame Survey of 2020 gleaning or walking surveys were acknowledged yet not included with monitored data. The document estimates that around the island of Zanzibar 14,965 were foot fishers out of which 6,708 were males and 8,257 were women, but that there could be many more especially if children were to be included. The Zanzibar Fisheries Frame Survey 2020 states that invertebrate gleaning is a SSF of lesser importance in comparison to vessel-based SSF (DFD 2020).

The three main management concerns in the local SSF mentioned at the focus session were overfishing in both gleaning and co-occurring SSF, overall poor fisheries management, and illegal fishing. Illegal fishing mentioned occurred within the present multi-gear fisheries and not the gleaning fishery. The mentioned illegal fishing activities were illegal mesh sizes in fishing nets, poison fishing, and spearfishing (Fig. 6). The participants in the focus group reported that the fisheries committee (local management) were ineffective in monitoring illegal fishing or in taking action when illegal fishing was reported. Participants themselves confessed having a lack of desire of reporting illegal fishing in solidarity with understanding the hardships that lead to illegal fishing. In addition, participants noted that local management lacks important resources, boats and financial support for effective monitoring of the SSF. Managers of the local fisheries were not based at Unguja Ukuu and need to travel far distances to monitor the SSF of Unguja Ukuu.

## Discussion

### Social-ecological interactions and outcomes of the gleaning fishery at Unguja Ukuu

Drawing on the different elements of this study, we found two negative social-ecological outcomes from our investigation of the gleaning fishery in Unguja Ukuu. Our study confirms the intertwined nature of the social and ecological components of the gleaning system at Unguja Ukuu. The two negative social-ecological outcomes discussed are changes in gleaned catch over time and a degrading intertidal zone. Both of these two social-ecological outcomes are important to understand the complex dynamics of gleaning fisheries for their management at Unguja Ukuu, around the island of Unguja, Zanzibar, and in other coastal areas where gleaning occurs.

Results from our catch landing surveys, household surveys, and focus group sessions discovered that certain gleaned species are becoming rare within the gleaned catch. Gleaners present at the focus session all agreed that economically important bivalves (*Modiolus* spp*.*) and gastropods (*Strombus* spp*.*) have become increasingly rare and these findings were paralleled with low abundances of both genera in our catch landing assessments (Supplementary information 1, Table S4, S5, S6). Other studies on the island of Zanzibar reflect similar findings to the gleaning fishery of Unguja Ukuu with perceived rapid changes in the catch (Crawford et al. 2010; Nordlund et al. 2010; Fröcklin et al. 2014). In the north of the island of Unguja, Zanzibar, a decline in catch per unit effort from 3 to 5 kg/per hour/ person to 2 kg/per hour/ person was reported within the last 30 years within one gleaning fishery (Nordlund et al. 2010). Yet, this study noted that the total number of species within the catch did not change compared to prior times (Nordlund et al. 2010). While another study of gleaning from Chwaka bay in Zanzibar indicated that invertebrate compositions changed significantly within a 5-year timeframe with consistent gleaning pressures upon the local intertidal (Fröcklin et al. 2014). This study from Chwaka bay reported that the composition of gleaned invertebrates in the catch was shared more evenly with other invertebrate species while our study was dominated by one species, the cockle (*Anadara antiquata*) (Fröcklin et al. 2014). Similar to this study of Unguja Ukuu the study of Chwaka bay found that economically important bivalves and gastropods have become rare within the catch (Fröcklin et al. 2014).

One noted behavior of the gleaners at Unguja Ukuu was the “take everything” practice while gleaning; referring to all invertebrates that can be sold, eaten, or used in the household in some other way. A similar “take everything” strategy in gleaning has been reported from other areas of the Indo-Pacific as well (Kyle et al. 1997; Nordlund et al. 2010; Fröcklin et al. 2014; Furkon et al. 2020). Our investigation showed a high species richness of 44 species within 29 gleaner landing surveys, but the catch composition was dominated by one cockle species (*A. antiquata*). Near our study site on the peninsula of Fumba the cockle (*A. antiquata*) was the most commonly collected invertebrate and this dominance of the cockle has remained so over time (Crawford et al. 2010). This indicates that *A. antiquata* has remained an important species for gleaning within the Menai Bay Conservation Area. Yet, at Unguja Ukuu it is perceived that gleaning efforts need to be increased to collect similar catches to prior times. Potentially, the high diversity within the catch in our study shows that gleaners make up for the lack of availability of preferential invertebrates by collecting all invertebrates that have any form of value. One result to note is the low abundance of eight octopus recorded in our catch landings (Supplementary information 1, Table S6), as octopus is commonly targeted during gleaning to be used for both food and income in the Western Indian Ocean (Fröcklin et al. 2014; Drury O’Neill et al. 2018). Possible explanations for the low abundance of octopus in this study could be due to seasonality, localized weather, or due to the availability of octopus within the gleaned coastal zone (Grantham et al. 2020).

Within the ecological survey of the intertidal at Unguja Ukuu, we found that the sea urchin (*Echinometra mathaei*) was highly abundant (Supplementary information 1, Table S4, S5). The highly abundant sea urchin (*E. mathaei*) within the intertidal seagrass of Unguja Ukuu could be a bioindicator of environmental degradation within the local intertidal zone. Within the WIO region, high abundances of these sea urchins (*E. mathaei*) are associated with environmental degradation from unsustainable fishing and coastal mismanagement (McClanahan and Shafir 1990; McClanahan and Mutere 1994; McClanahan 1994). Based on the high abundance of the sea urchin (*E. mathaei*) our study suggests that the composition of invertebrates within the intertidal of Unguja Ukuu is degrading and that the intertidal zone might be under multiple pressures.

Intensive gleaning fisheries similar to the one at Unguja Ukuu are associated with declines in intertidal invertebrates and invertebrate gleaning often damages the intertidal zone (Nordlund et al. 2010; Nordlund and Gullström 2013; Fröcklin et al. 2014; Alati et al. 2020). The impacts of invertebrate gleaning onto the intertidal zone and the multiple perceived threats from this study on the invertebrate community need to be better understood. For example, considering the removal of invertebrates such as filter feeders (e.g. bivalves) may reduce nutrient cycling, water clarity, and the regulation of phytoplankton while the removal of grazers (e.g. gastropods) may result in the loss of entrapped primary production (Lonsdale et al. 2009; Fong et al. 2018; Cranford 2019). Unregulated extraction of invertebrates that contribute to the functionality of the coastal ecosystems may degrade a plurality of ecosystem services. Additionally, local pressures from the co-occurring vessel-based SSF may impact the trophic cascade and ultimately the composition and availability of invertebrate species (McClanahan and Shafir 1990; McClanahan and Mutere 1994; McClanahan 1994). Shifts in the regional climate within the Indo-Pacific may result in increased frequency and magnitude of extreme weather events and increases in sea surface temperature which may stress or disturb the intertidal ecosystems (Grech et al. 2012).

### Considerations for future social-ecological systems analysis of SSF

The different research methods used in this study namely the ecological surveys, catch assessments, interviews with gleaners, household surveys, focus group interviews and analyzing the governance resulted in an understanding of the gleaner fishery at Unguja Ukuu as a SES. Thus, we recommend future studies of SSF to integrate several methods both social and ecological; to gain a deeper understanding of the intertwined nature of a SES. This study investigated the SES of invertebrate gleaning only at one point in time, but SES are dynamic with society and nature interacting, changing, and co-evolving over time (Ostrom 2009). Therefore, time is important to consider in an analysis of any SES. In our study, the invertebrates of the intertidal and gleaner landings were surveyed during spring low tide which may have impacted the species recorded within our study. Within the context of coastal East Africa, fishery resources inhabiting intertidal ecosystems may have seasonal, yearly, or decadal variations that could be both natural and human-induced (Nordlund et al. 2010; Alonso Aller et al. 2019). Additionally, fishing practices and social norms within a SSF may change over time based on the social-economic setting and context (Cinner 2009, 2011, 2009).

In our investigation, Local Ecological Knowledge (LEK) was obtained from several sources in our field surveys (including gleaning landing surveys, household surveys, and a focus session) to better understand the interactions and outcomes of the SES. While the use of LEK is controversial due to memories bias and shifting baseline (Papworth et al. 2009; Daw et al. 2011) this interview method was performed since perceptions of local fishers can be a starting point to investigate data-poor fisheries (Johannes et al. 2000) as in the case of the invertebrate gleaning fishery in Unguja Ukuu. LEK in similar gleaning fisheries of Kenya has been used to recreate recent historic occurrences and distributions of intertidal mollusks (Alati et al. 2020). Similarly in our study, we required LEK to better understand gleaning as a SES and the outcomes of the interacting components.

### Invertebrate gleaning: A broader perspective

There is no doubt that gleaning is commonplace and important for many women in Zanzibar (de la Torre-Castro and Rönnbäck 2004; Crawford et al. 2010; Nordlund et al. 2010; Fröcklin et al. 2014), but in the Fisheries Act of 2010, invertebrate gleaning is not explicitly defined as a SSF. Furthermore, invertebrate gleaning fisheries are data-poor with little to no baseline data on the catch and effort in Zanzibar (Nordlund et al. 2010; Fröcklin et al. 2014; Rehren et al. 2020). Just north of Unguja Ukuu, a study found that gleaning was widespread and was a complicated SSF to manage with the attempts of no-take zones facing encroachment from the local gleaning community (Crawford et al. 2010). Also on a global scale, invertebrate fisheries are generally receiving less attention than other SSF targeting finfish and commercial fisheries, both in terms of assessment and regulations, showing the need for enhanced management attention of invertebrate fisheries (Anderson et al. 2011; Harper et al. 2013; Silas et al. 2022). Invertebrate gleaning fisheries in rural coastal areas face the challenges of not being recognized as a true fishery and may receive less attention because the fishers are often women and the targeted species are invertebrates.

This female-dominated fishery, important for income and food, is also of social and cultural value to the gleaners and the community at large (Nordlund et al. 2010; Grantham et al. 2020). In Unguja Ukuu, some of the gleaners were gleaning as a social activity with their peers and we also found that invertebrate gleaning holds a cultural value to the local community. For example, we found that gleaned invertebrates and their profits were commonly used to support cultural activities (weddings and funerals) and used as cultural ornaments. During several household surveys, we found that gleaned invertebrates species (*Moneteria* spp.) were used as playing pieces in the traditional game, Bao (Fig. 5). A study from northern Zanzibar in 2007 showed that 24% of the interviewed gleaners felt that gleaning was also a social activity while the majority 76% said it is simply work (Nordlund et al. 2010). In another study of an invertebrate gleaning fishery on the island of Timor–Leste, the social aspects or non-material values of invertebrate gleaning were deemed important for human wellbeing and were considered an additional reason to glean (Grantham et al. 2020).

## Conclusion

The SES framework was a practical analytical tool for investigating the invertebrate gleaning fishery at Unguja Ukuu. The use of the four components of the SES framework assisted in guiding our interdisciplinary study. This study embraced a mixed method approach to investigate SSF using both ecological and social surveys. We suggest to other researchers and local managers of SSF to utilize different methods to develop the understanding of their SSF in focus. Potentially, if this investigation included just social or ecological methods then we might have limited our findings. The different methods when analyzed together revealed two main social-ecological outcomes; a change in gleaned catch and a degrading intertidal zone. These two changes or outcomes of the SSF were perceived to derive from overharvesting by invertebrate gleaners and from outside pressures onto the intertidal zone. Our investigation found that gleaning for invertebrates has become less profitable, yet the majority of the households at Unguja Ukuu are becoming more reliant upon gleaning as a source of income and food.

Invertebrate gleaning is still considered an invisible fishery which is often ignored within fisheries management strategies and this study aims to bring more awareness to the importance of this SSF (Kleiber et al. 2014; Rehren et al. 2020). Considering the management and governance of invertebrate gleaning we conclude with the following recommendations: Invertebrate gleaning should be explicitly defined and incorporated within the law and policy. Baseline data for how many are gleaning along with what and how much is being landed would be important to gain a deeper understating of this SSF. Establishing monitoring of gleaning, in the same manner as for fin-fisheries, could enable detection of declines but also develop a better understanding of the environmental consequence of invertebrate gleaning. Furthermore, long term monitoring may lead to an understanding of how to manage invertebrate gleaning and what social-ecological interactions need to be regulated. Considering the management of both invertebrate gleaning and co-occurring SSF we suggest involving the local community. The integration of the local communities, i.e. the stakeholders directly dependent on these resources, into natural resource management has been reported to reduce illegal activities and increase sustainable resource usage (Verheij et al. 2004; Epstein 2017). At the study site Unguja Ukuu, the alternative livelihood sea cucumber farm and the “no-take zone” of bivalves were not successful, as both attempts failed due to the lack of the local community championing these undertakings. Even though, we still suggest engaging the local community is crucial for improved management of invertebrate gleaning and co-occurring SSF.

## Supplementary Information

Below is the link to the electronic supplementary material.

Supplementary file1 (PDF 2166 kb)
