# Supplementary material for: Social-ecological system analysis of an invertebrate gleaning fishery on the island of Unguja, Zanzibar
Source: Ambio. 2022 Aug 9;52(1):140–54. doi: 10.1007/s13280-022-01769-1 (PMC9666602; doi:10.1007/s13280-022-01769-1)
Supplement: Supplementary file 1 — Supplementary file1 (PDF 2166 kb) [file 13280_2022_1769_MOESM1_ESM.pdf]

Ambio

Electronic Supplementary Material

This supplementary material has not been peer reviewed.

Social-ecological system analysis of an invertebrate gleaning fishery on the island of Zanzibar

Authors:
Johann Stiepani[1], Narriman Jiddawi[2], Lina Mtwana Nordlund[1]
1. Natural Resources and Sustainable Development, NRHU Department of Earth Sciences, Uppsala University, Campus Gotland, Sweden.
2. Institute of Marine Sciences, University of Dar Es Salaam, Tanzania
Corresponding author: Johann Stiepani
Email: johann.stiepani@geo.uu.se

Table S1. Gleaner landing survey

1. Do you agree to participate in this study?

2. How old are you?

3. Fill in the gender (man/woman) First, we have some questions about your gleaning habits

4. Why do you glean? (multiple choice: For extra income, Main income, For food, To get more varied diet, To collect bait, Curio, Nothing else to do, Social, Other)

5. What do you typically do with your catch? (multiple choice: Market in the village, Town market, International market, Home consumption, For curio trade, To make art or jewellery, Bait, Other)

6. How many days a month do you glean?

7. For how many hours during one day do you typically glean?

8. Do you use any equipment when gleaning? (multiple choice: Hands, Rock, Knife, Wooden stick, Metal stick, Rake, Trap, Net, Mosquito net, Hand lines, Fishing rod, Boat to go to gleaning site, Other)

9. What equipment do you typically use? (multiple choice: Hands, Rock, Knife, Wooden stick, Metal stick, Rake, Trap, Net, Mosquito net, Hand lines, Fishing rod, Boat to go to gleaning site, Other

10. What is your favourite habitat to glean in? (multiple choice: Coral reef, Deep water, Mangrove, Mud, Sand, Seagrass, Algae, Other)

11. In which habitat do you get the most valuable catch (in monetary terms)? (multiple choice: Coral reef, Deep water, Mangrove, Mud, Sand, Seagrass, Algae, Other) We also have some questions of the gleaning you have done today.

12. For how many hours have you been gleaning today?

13. In which habitat/-s have you been gleaning today? (multiple choice: Coral reef, Deep water, Mangrove, Mud, Sand, Seagrass, Algae, Other)

14. Have you been gleaning in the seagrass today? Y/N

15. (if gleaned in seagrass) How many hours did you spend in the seagrass today?

16. Is your catch today, larger, smaller, or the same size as you typically catch? Multiple choice: Much smaller, Smaller, Same size, Larger, Much larger)

17. What will you do with your catch today? (multiple choice: Market in the village, Town market, International market, Home consumption, For curio trade, To make art or jewellery, Bait, Other)

18. (if they sell) How much money will you get for this catch today (on an average day)? Local currency or USD?

19. How many people have contributed to the collection of your catch? Any additional comments from interviewee Any additional comment from team interviewing If you have not been able to weigh and look at the catch during the interview, please do so now.

20. May I please weigh your catch? Weigh catch and record the weight of the whole catch in kilo (kg). (100g=0.1kg)

21. May I please look a bit closer at your catch and spread it on a plastic sheet and take pictures? Investigate the catch, preferably on a plastic sheet, take photos and identify species the catch to lowest taxonomical level possible. Start recording the most common and "valuable"species. Take a picture of the whole catch. Remember to include a ruler or something with a known length (and define that

length here above in the form) in all photos. Include your ID for the catch (e.g. a note with date and time you started the interview)

Table S2. Household survey focused on food security

Introductory questions

1. Village/Sex/Age/Education
2. How many years have you performed seagrass gleaning?
3. Do you perform gleaning in any other ecosystem? (which one?)
4. Which ecosystem do you prefer? (and why?)
5. What is the main reason for gleaning in seagrass?
    a. Income
    b. Food for the household
    c. Both
    d. Other, please specify

Availability

6. What kind of invertebrates do you collect in the seagrass?
7. Has the variety changed over time?
    a. Yes. If yes, please describe
    b. No
    c. I do not know
8. During the years you have performed seagrass gleaning in Unguja Ukku, do you think that the catch has
    a. increased? Why do you think it has increased?
    b. decreased? Why do you think it has decreased?
    c. remained the same?
    d. I do not know
9. During the years you have performed seagrass gleaning in Unguja Ukku, do you think that the number of people that perform gleaning has
    a. increased? Why do you think it has increased?
    b. decreased? Why do you think it has decreased?
    c. remained the same?
    d. I do not know
10. According to your knowledge, approximately how many people in Unguja Ukku perform seagrass gleaning?

Accessibility

11. How long do you have to walk during collection? (Hours)
12. If you could no longer access this area, what would you do?
    a. Glean in another place
    b. Stop to do gleaning
    c. Try to do another activity (which one?)
    d. I do not know
13. Is it difficult or dangerous to do seagrass gleaning here?
    a. Yes. If yes, please specify why
    b. No
    c. I have no opinion
14. In your opinion, in which seagrass area do you find the best invertebrates? (e.g. best quality, biggest catch, best value)
15. Can you access that area?
    a. Yes. If yes, please specify how
    b. No. If no, please specify why
    c. I do not know
16. What protein sources (foods) do you have access to?

a. Beef (every day/a few days a week/once a week/a few times a month/other)
b. Lamb/goat (every day/a few days a week/once a week/a few times a month/other)
c. Chicken/other poultry (every day/a few days a week/once a week/a few times a month/other)
d. Eggs (every day/a few days a week/once a week/a few times a month/other)
e. Fish (finfish) (every day/a few days a week/once a week/a few times a month/other)
f. Invertebrates (every day/a few days a week/once a week/a few times a month/other)
g. Legumes, e.g. beans, lentils, peas, soy foods, peanuts - (every day/a few days a week/once a week/a few times a month/other)
h. Tree nuts, e.g. cashew nuts, (every day/a few days a week/once a week/a few times a month/other)
i. Bush meat (every day/a few days a week/once a week/a few times a month/other)
j. Other

17. Are there any alternative livelihoods/incomes in your household?
    a. Yes, If yes, please specify which (and who is performing this other livelihood: you, husband, children)
    b. No

Utilisation and nutrition

18. What do you do with the catch?
    a. Eat
    b. Sell
    c. Exchange for other foods
    d. Other, please specify
19. If you sell the invertebrate / their by -product, what do you do with the income?
20. How many people do you need to feed in your household?
21. Can you store your catch and eat it later?
    a. Yes
    b. No
    c. I do not know
22. If you can store the invertebrates from seagrass, how is it done?
    a. Smoked
    b. Salted
    c. Sun dried
    d. Boiled
    e. Cold storage: ice/fridge/freezer?
    f. Other

Stability over time

23. Does the catch vary over the northeast monsoon (kaskazi) and southeast monsoon (kusi)?
24. How many days a month do you glean in seagrass?
25. How many days a month do you glean in another ecosystem?
26. According to you opinion, is there anything that threatens the provision of seagrass invertebrates?
    a. Industry
    b. Tourism
    c. Too many gleaners
    d. Plastic
    e. Waste water
    f. Climatic conditions/weather
    g. Ecological changes that decrease the invertebrates
    h. Other. Please specify what
    i. I do not know
27. Is there anything one can do to protect the invertebrates for future generations?
    a. Yes. If yes, how do you think it should be protected?

    b. No. If no, why don't you think it cant be protected?
    c. No opinion/I do no know
28. How important do you think this seagrass gleaning activity is to provide food/protein to your household (either by your own consumption, or through providing income to buy other food/protein)
    a. Very important
    b. Important
    c. Not so important
    d. Not important at all

Table S3. Focus session

Section 1 – Changes to gleaning over time

- Have your experiences with gleaning changed over time?
- Are there differences in the species you collected when you first started, to now?
- Have you noticed any particular species disappearing?
- Do you target any kinds of animals in particular? Are there certain animals which you can gain a cash income from?
- How important is gleaning for both your household income, and for providing food?
- Do you feel that gleaning provides you with a good livelihood?
- Has there been changes in the numbers of men gleaning?

Section 2 – Gleaning habitats

- Have you noticed any changes in the coastal areas where you glean?
- Which of these habitats do you feel are important for coastal areas? Please give some reasons.
- What kind of changes have you noticed in the seagrass over time?
- What do you feel has driven these changes?

Section 3 – Gleaning Management

- Do you feel that gleaning gets enough attention from coastal managers?
- Have you noticed any attempts of management with gleaning, what were they and how helpful do you think they were?
- What do you think could be done to help make gleaning activities more sustainable, so that there's enough animals to collect into the future?
- What resources do you think could help with this?
- What problems do you see associated with developing a management strategy for gleaning?
- Out of the following management strategies, which do you feel would be the most achievable, and helpful?

I. Minimum catch size limits
II. Limitations on the most damaging types of equipment
III. Local beach recorders who make records of catches
IV. Committees made up of gleaners to discuss issues and problem solving
V. Education workshops to learn more about the animals, and coastal ecosystems
VI. Limits on total allowable catch
VII. Temporal and spatial closures of overharvested sites

Table S4. Species list from the ecological survey. Including total abundance of each invertebrate in the study. Invertebrate species densities and standard deviations are listed for the fifteen unvegetated transects and eighteen vegetated transects at the spatial resolution of 1 m^2 with average dissimilarity (SIMPER test) of invertebrate densities between unvegetated transects and vegetated transects. Both the small and large invertebrate sampled in the ecological assessment densities are presented in this table, aside each species name the following letter represent S for being sampled as a small invertebrate, L for being samples as a large invertebrate, and S+L if species were sampled as both small and large invertebrates. Significant differences for invertebrate species densities are given as *p<0.05 (Mann – Whitney U test).

Family and Superfamily	Species	Total abundance	Invertebrate density Unvegetated	Invertebrate density Vegetated	Average dissimilarity
Alpheoidea	*Alpheus sp.* (S)	1	0	4 ± 0.92	0.36
Arcoidea	*Anadara antiquata* (S+L)	4	0	8.02 ± 1.26	1.91
Buccinoidea	*Nassarius sp.* (S)	1	0	4 ± 0.92	2.39
Calappoidea	*Calapa hepatica* (S)	1	0	4 ± 0.92	0.45
Cassiopeidae	*Cassiopea andromeda* (L)	13	0.05 ± 0.009	0.08 ± 0.01	0.05
Cypraeoidea	*Cypea tigris* (L)	2	0	0.02 ± 0.005	0.01
	Cypraea annulus (S)	9	8 ± 2.0	28 ± 3.02	8.35
Edwardsiidae	*Edwardsianthus spp.* (S)	855	11.85 ± 2.0	4.68 ± 1.01	5.74
Eriphioidea	*Eriphia smithii* (L)	1	0.01 ± 0.002	0	0.01
	Epixanthus frontalis (L)	3	0	0.03±0.01	0.05
Gonodactyloidea	*Odontodactylus sp.* (L)	1	0.01 ± 0.002	0	0.001
Holothuriidae	*Holothuria spp.* (S+L)	23	0.1 ± 0.017	20.11 ± 4.59	2.85
Mytiloidea	*Modiolus philippinarum* (S+L)	14	0.05± 0.01	12.06±2.0	4.6
Naticoidea	*Nautica gualteriana* (S)	1	0	4 ± 0.92	1.47
Neritoidea	*Nerita albicilla* (S)	2	8 ± 2.0	0	0.83
Ocypodoidea	*Chaenostoma boscii* (S)	4	16 ± 3.09	0	3.85
	Macrophthalmus milloti (S)	1	0	4± 0.92	1.29
Odontophora	*Echinometra mathaei* (L)	817	1.3 ± 0.21 *	6.87 ± 0.66 *	4.53
Ophiocomoidea	*Ophiocoma sp.*(S)	2	0	8 ± 1.83	1.47
Paguroidea	*Clibanarius spp.* (S+L)	154	356.01 ± 45.59	252 ± 29.07	41.35
Pinnoidea	*Pinna muricata* (L)	135	0.39 ± 0.05	0.96 ± 0.08	0.9
	Atrina vexillum (S+L)	6	0	20.01 ± 4.58	2.27

Portunoidea	*Portunus pelagicus* (L)	6	0.01 ± 0.002	0.05 ± 0.006	0.02
	Thalamita poissonii (L)	1	0	0.01 ± 0.002	0.001
Pterioidea	*Pinctada margaritifera* (L)	36	0.09 ± 0.2	0.27 ± 0.03	0.39
	Isognomon isognomon (L)	6	0	0.06 ± 0.01	0.01
Synaptidae	*Synapta spp.* (L)	49	0.05 ± 0.009	0.44 ± 0.05	0.32
Temnopleuridae	*Salmacis bicolor* (L)	3	0	0.03±0.01	0.04
Trochoidea	*Trochus spp.* (S+L)	18	0.08 ± 0.01	28.0 ± 4.45	5.44
	Total	2,170			

Table S5. Abundance data for invertebrates separated as large invertebrates (L) and small invertebrates (S) from the ecological survey of the intertidal

	Clibanarius spp.	*Cypraea annulus*	*Trochus spp*	*Modiolus philippinarum*	*Alpheus sp.*	*Chaenostoma boscii*	*Holothuria spp.*	*Nassarius spp.*	*Calapa hepatica*
unvegetated	0	0	0	0	0	0	0	0	0
unvegetated	2	0	0	0	0	0	0	0	0
unvegetated	24	0	0	0	0	0	0	0	0
seagrass	10	0	0	0	0	0	0	0	0
seagrass	5	1	0	0	0	0	0	0	0
seagrass	30	0	0	0	0	0	0	0	0
seagrass	0	1	0	0	0	0	0	0	0
seagrass	10	1	0	0	0	0	0	0	0
seagrass	0	0	0	0	0	0	0	1	0
seagrass	0	0	4	0	0	0	0	0	0
seagrass	0	0	3	0	0	0	3	0	0
seagrass	0	0	1	0	0	0	2	0	1
unvegetated	3	0	0	0	0	0	0	0	0
unvegetated	0	0	0	0	0	3	0	0	0
unvegetated	1	0	0	0	0	0	0	0	0
seagrass	0	0	0	0	0	0	0	0	0
seagrass	0	0	0	2	0	0	0	0	0
seagrass	0	1	0	0	0	0	0	0	0
unvegetated	4	0	0	0	0	0	0	0	0
unvegetated	0	0	0	0	0	0	0	0	0
unvegetated	0	0	0	0	0	0	0	0	0
unvegetated	0	0	0	0	0	0	0	0	0
unvegetated	1	0	0	0	0	0	0	0	0
unvegetated	1	2	0	0	0	0	0	0	0
seagrass	0	0	0	0	0	0	0	0	0
seagrass	0	0	2	0	0	0	0	0	0
seagrass	0	0	0	0	0	0	0	0	0
unvegetated	11	0	0	0	1	0	0	0	0
unvegetated	8	0	0	0	0	1	0	0	0
unvegetated	41	0	0	0	0	0	0	0	0
seagrass	0	0	0	1	0	0	0	0	0

seagrass	0	3	0	0	0	0	0	0	0
seagrass	0	0	0	0	0	0	0	0	0
	S	S	S	S	S	S	S	S	S

Nerita albicilla	*Anadara antiquata*	*Atrina vexillum*	*Macrophthalmus milloti*	*Edwardsianthus spp.*	*Clypeomorus bifasciatus*	*Nautica gualteriana*	*Ophiocoma sp.*
0	0	0	0	0	0	0	0
0	0	0	0	0	0	0	0
0	0	0	0	0	0	0	0
2	0	0	0	0	0	0	0
0	0	0	0	0	0	0	0
0	0	0	0	0	0	0	0
0	0	0	0	0	0	1	0
0	0	0	0	0	0	0	0
0	0	0	0	0	0	0	0
0	0	0	0	0	0	0	0
0	0	0	0	0	0	0	0
0	1	5	0	0	0	0	0
0	0	0	0	0	0	0	2
0	0	0	0	0	0	0	0
0	0	0	0	0	0	0	0
0	0	0	0	2	0	0	0
0	0	0	0	0	0	0	0
0	1	0	0	0	0	0	0
0	0	0	0	0	0	0	0
0	0	0	0	0	0	0	0
0	0	0	0	0	0	0	0
0	0	0	0	0	0	0	0
0	0	0	1	0	0	0	0
0	0	0	0	0	0	0	0
0	0	0	0	0	0	0	0
0	0	0	0	0	0	0	0
0	0	0	0	0	0	0	0
0	0	0	0	0	0	0	0
0	0	0	0	0	0	0	0
0	0	0	0	0	0	0	0
0	0	0	0	0	0	0	0
0	0	0	0	0	0	0	0
0	0	0	0	0	0	0	0
S	S	S	S	S	S	S	S

Portunus pelagius	*Echinometra mathaei*	*Cassiopea andromeda*	*Pinna muricata*	*Thalamita poissoni*	*Eriphia smithi*	*Holothuria spp*	*Cypea tigris*	*Trochus spp.*	*Pinctada margaritifera*
0	0	0	0	0	0	0	0	0	0
0	0	0	0	0	0	0	0	0	0
0	0	0	0	0	0	0	0	0	0
1	0	0	0	0	0	0	0	0	0
2	0	0	0	0	0	0	0	0	0
0	5	1	0	0	0	0	0	0	0
1	0	0	0	0	0	0	0	0	0
0	1	1	0	0	0	1	0	0	0
0	3	0	0	0	0	1	0	0	0
0	6	0	1	0	0	0	0	0	0
0	7	0	8	0	0	1	0	0	0
0	19	0	3	0	0	3	0	0	0
0	0	0	0	1	0	0	0	0	0
0	0	0	0	0	0	0	0	0	0
0	0	0	0	0	1	0	0	0	0
0	22	0	5	0	0	5	0	0	0
1	18	0	9	0	0	4	2	0	0
0	12	1	5	0	0	1	0	0	0
0	3	5	3	0	0	0	0	0	0
0	3	0	0	0	0	0	0	0	0
0	1	3	4	0	0	0	0	0	0
0	0	0	0	0	0	0	0	0	0
0	10	0	2	0	0	3	0	0	0
0	8	2	9	0	0	2	0	0	0
0	86	0	19	0	0	0	0	8	9
1	96	0	10	0	0	0	0	0	0
0	49	0	30	0	0	0	0	0	8
0	0	0	0	0	0	0	0	0	0
0	0	0	0	0	0	0	0	0	0
0	0	0	0	0	0	0	0	0	0
0	238	0	1	0	0	0	0	0	8
0	182	0	16	0	0	0	0	0	0
0	48	0	10	0	0	0	0	0	11
L	L	L	L	L	L	L	L	L	L

Epixanthus frontalis	*Atrina vexillum*	*Anadara antiquata*	*Isognomon isognomon*	*Clibanarius spp.*	*Edwardsianthus spp.*	*Synapta spp.*	*Modiolus philippinarum*	*Salmacis bicolor*	*Epixanthus frontalis*
0	0	0	0	0	0	0	0	0	0
0	0	0	0	0	0	0	0	0	0
0	0	0	0	0	0	0	0	0	0
0	0	0	0	0	0	0	0	0	0
0	0	0	0	0	0	0	0	0	0
0	0	0	0	0	0	0	0	0	0

0	0	0	0	0	0	0	0	0	0
0	0	0	0	1	0	0	0	0	0
0	0	0	0	0	1	0	0	0	0
0	0	0	0	0	0	0	0	0	0
0	1	0	0	0	5	0	0	0	0
0	0	0	0	0	0	0	0	0	0
0	0	0	0	0	0	0	0	0	0
0	0	0	0	0	0	0	0	0	0
0	0	0	0	0	0	0	0	0	0
0	0	0	0	0	0	0	0	0	0
0	0	0	0	0	0	0	0	0	0
0	0	0	0	1	0	0	0	0	0
0	0	0	0	0	442	1	0	0	0
0	0	0	0	0	0	0	0	0	0
0	0	0	0	0	32	0	0	0	0
0	0	0	0	0	160	2	0	0	0
0	0	0	0	0	126	0	0	0	0
0	0	0	0	0	39	0	0	0	0
0	0	0	0	0	28	3	5	0	0
0	0	0	0	0	0	9	6	1	0
0	0	0	0	0	0	6	0	2	3
0	0	0	0	0	0	0	0	0	0
0	0	0	0	0	0	0	0	0	0
0	0	0	0	0	0	0	0	0	0
0	0	2	0	0	20	20	0	0	0
0	0	0	6	0	0	8	0	0	0
0	0	0	0	0	0	0	0	0	0
L	L	L	L	L	L	L	L	L	L

Table S6.Total abundance and composition (%) of invertebrate species recorded during the gleaning landing assessment

Family and Superfamily	Species	Abundance	Composition (%)
Arcoidea	*Anadara antiquata*	1,067	50.43
Buccinoidea	*Volema pyrum*	7	0.33
	Pleuroploca trapezium	35	1.65
Carapidae	*Carapidae sp.*	1	0.05
Cardioidea	*Cardiidae spp.*	114	5.39
Carditoidea	*Carditidae spp.*	15	0.71
Cerithioidea	*Clypeomorus bifasciatus*	1	0.05
Conoidea	*Conus bandanus*	1	0.05
	Conus rattus	3	0.14
	Conus sp. 1	9	0.43
	Conus sp. 2	1	0.05
Cypraeoidea	*Monetaria annulus*	63	2.98

	Erronea caurica	1	0.05
	Cypraea tigris	42	1.98
	Lyncina vitellus	3	0.14
Diodontidae	*Diodon hystrix*	2	0.09
Holothuriidae	*Holothuria scabra*	6	0.28
	Holothuria atra	1	0.05
Loliginidae	*Sepioteuthis lessoniana*	5	0.24
Mactroidea	*Mactra ovalina*	10	0.47
Muricoidea	*Chicoreus ramosus*	153	7.23
Mytiloidea	*Modiolus philippinarum*	14	0.66
Naticoidea	*Polinices mammilla*	2	0.09
Neritoidea	*Nerita albicilla*	3	0.14
Octopodoidea	*Octopus cyanea*	8	0.38
Ostraciidae	*Lactoria cornuta*	1	0.05
Paguroidea	*Paguroidea spp.*	6	0.28
Pinnoidea	*Pinna muricata*	24	1.13
	Atrina vexillum	24	1.13
Platycephalidae	*Platycephalus indicus*	4	0.19
Plotosidae	*Paraplotosus sp.*	1	0.05
Portunoidea	*Portunus pelagicus*	113	5.34
Pterioidea	*Isognomon isognomon*	93	4.40
	Pinctada margaritifera	147	6.95
Serranidae	*Epinephelus sp.*	1	0.05
Stromboidea	*Strombus gibberulus*	3	0.14
Synanceiidae	*Synanceia verrucosa*	1	0.05
Tellinoidea	*Semelidae sp.*	1	0.05
Trochoidea	*Trochidae spp.*	101	4.77
Turbinelloidea	*Vasum rhinoceros*	12	0.57
Veneroidea	*Gafrarium sp.*	3	0.14
	Pitar hebraeus	1	0.05
	Veneridae sp. 1	8	0.38
	Veneridae sp. 2	5	0.24
	Total	2,116	

IPSN Research protocol

Ecological surveys of seagrasses and invertebrates

IPSN ECOLOGICAL SURVEYS OF SEAGRASSES AND INVERTEBRATES

Summary

Please read through the entire manual before starting.
In order to understand the role of seagrasses in supporting invertebrate fisheries we need ecological background data on the seagrass habitat and the invertebrate community. To gather the ecological background data, IPSN participants are asked to conduct a seagrass and invertebrate survey using this research protocol (Ecological surveys of seagrasses and invertebrates) in your IPSN field location where gleaning occurs (if possible).

In specific, the IPSN ecological survey of seagrasses and invertebrates has the following aims and objectives:

1. Assess the species, density and condition of the seagrass in the site.
2. Quantify the invertebrate community present in both quadrates and in belt transects in both the seagrass and un-vegetated site.

For this research protocol, two sites are needed in your location: one *seagrass site* (an area where seagrasses dominate and that is gleaned, if possible) and one *un-vegetated site* (bare sediment without seagrass, algae and coral). The un-vegetated site will function as a reference or 'control' area, to be compared with the seagrass site. Comparisons of the invertebrate community in the seagrass and the un-vegetated site (reference area) will be done, to estimate the role of seagrass for secondary (invertebrate) production.

A seagrass survey will be conducted in the seagrass site to assess the characteristics of the seagrass. This is done by collecting data on multiple seagrass variables in replicated quadrats along transects, following the SeagrassWatch method.

Surveys of both small and large invertebrates will be conducted in both the seagrass site and the un-vegetated site. Large invertebrates (>5cm) are sampled along each transect. Small invertebrates (>1cm) are sampled at replicated points along each transect.

There are also IPSN training videos available on this link:
https://www.youtube.com/playlist?list=PLrdfk094tjkJCLWe5IxrtoRAuXArliRGC

<u>Your location</u>

The IPSN location is the coastal area where you will conduct your IPSN research and all different research protocols. The selection of location needs substantial consideration. Please attend to the *IPSN Location survey research protocol* to help you with your location decision, if you have not yet decided your IPSN location. Do not hesitate to contact the IPSN core team to discuss location choice.

Equipment needed for ecological surveys of seagrasses and invertebrates

- 50 m tape measure (preferably three of these)
- 6 (or 9) pegs, sticks or weights (to hold transect lines in place)
- 0.5 x 0.5m sampling quadrat
- 0.25 x 0.25m sampling quadrat

- 30 cm ruler (to measure seagrass canopy height)
- A 2.0 m long stick with a marking at 1.0 m (to aid in the sampling of large invertebrates along the transect)
- Android phone or tablet (with GeoODK and the research protocol installed) OR writing slate with water-proof paper & pencils
- GPS in your Android device OR a handheld GPS with spare batteries.
- Camera in your android device OR Camera (not required)
- Identification literature for invertebrates and seagrass – this can also be downloaded to your Android phone or tablet. Print and laminate the seagrass species identification keys.
- Suitable field clothes (wet boots, hat, sunglasses etc.)
- Battery pack for Android phone or tablet (not required)
- Waterproof bag or pouch (preferably transparent to be able to take pictures) that fits your Android device (and battery pack) OR camera and GPS (not required)
- Underwater viewer (e.g. bucket with glass bottom) or snorkelling gear if the meadow is subtidal
- Protective gloves (if available)
- Knife or sharp stick (if available)

STEP 1: Identifying suitable study sites at your location

First, two sampling sites needs to be identified; one seagrass site and one un-vegetated site (figure 1). These two sites should be of similar physical environment (e.g. same exposure level, relative water depth) and adjacent to each other.

Ideally, the sites should be intertidal (fully exposed during spring low tide) or shallow subtidal (<0.2m depth at spring low tide). In order to sample a shallow subtidal site the team may require the use of an underwater viewer (glass viewing box) or snorkelling gear.

Since sampling will be conducted during *spring low tide*, it is important to be aware of the tidal cycle in the area. Tide charts are available online, but if your location is far from the closest area reported in a tide chart, the timing of the low tide might deviate from the tide chart. The fishers in your location will also know the timings of the tides.

Site identification may require sending a member of the team on an initial fact-finding trip; do not rush into making site choices and discuss this as a team. Depending on your prior knowledge of the location, this fact-finding trip can be combined with data collection for other IPSN research protocols, such as habitat mapping and location survey. During this fact-finding trip it is also advisable to get an overview of which are the dominating seagrass and invertebrate species in the sites, to facilitate your sampling in the field.

Choosing the seagrass site

Identify an intertidal or shallow subtidal (<0.2m depth at low tide) seagrass meadow (seagrass-dominated area) where you have seen that people collect invertebrates by hand (gleaning), and within which you can easily fit a 50 x 50 m intertidal seagrass sampling area (see Fig 1).

The site needs to be within the meadow, at least 10m away from a meadow edge.

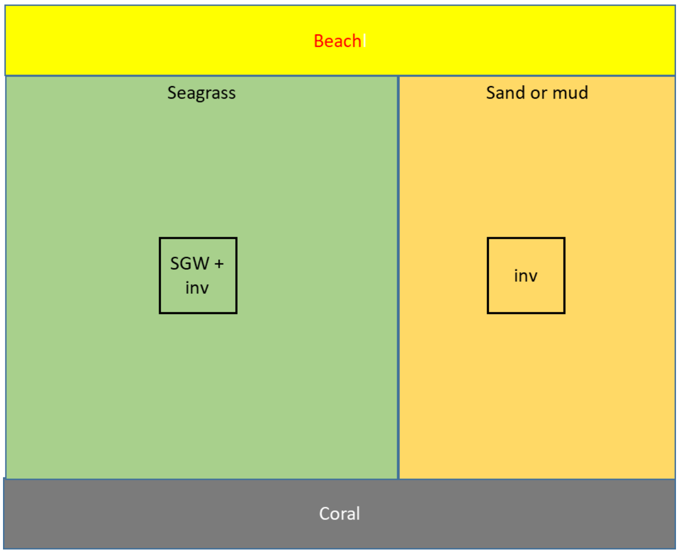

Figure 1. Seagrass and invertebrate sampling site layout.

Choosing the un-vegetated site

Then identify a nearby un-vegetated site, that will serve as a reference to your seagrass site. Ideally, this should have sand or mud bottom and no seagrass, macroalgae or coral, and be situated at the same distance from the shoreline as the seagrass site, and at the same depth, and that can also easily fit a 50 x 50 m sampling area. The un-vegetated site (reference area) should preferably be adjacent to the seagrass site.

Ideally, the un-vegetated site should have similar sediment type as in your seagrass site. For example in your seagrass site, the seagrass grows in mud, you should aim to choose an un-vegetated site dominated by mud. If the seagrass in your seagrass site grows in sand, choose an un-vegetated site dominated by sand.

In some locations it could be difficult to find a completely barren unvegetated site (a site with no seagrass at all, and that does not have any macroalgae growing on it). If this is the case, you can instead choose a site that has very low seagrass cover (less than 10% cover) or a site that has no seagrass but some macroalgae.

If you cannot find a suitable un-vegetated site at your location, please take photos of the sites available, and GPS position of the location, and contact the IPSN core team to discuss options.

STEP 2: PREPARATIONS FOR FIELD SAMPLING

If you are inexperienced with doing transect surveys, seagrass surveys, invertebrate identification, SeagrassWatch and/or you have never worked together as a team before, we strongly advice that you practise the whole (or part of the) sampling steps on land or at another site, before collecting the actual data for IPSN.

When you do the actual sampling, it may also be good to start with the sampling in the un-vegetated site, because it will have no or little seagrass and other vegetation. Therefore, the survey in the unvegetated area is likely to be easier and quicker.

Before you start sampling, assign clear tasks for all team members, and decide who is doing what, and in what order. For example, someone can be in charge of recording the data (using ODK collect or on paper) and another person in charge of invertebrate identification, estimating seagrass cover, etc.

Overview of sampling

Details of all three components of this sampling is described below this section.

1. All sampling should be undertaken during spring low-tide, at the same time and tide as invertebrate gleaning would take place.
Make sure to be at the site and prepare everything well in time before sampling starts. It takes time to set up the transects and you will want to start collecting data as soon as the tide is low enough.

2. Begin at the un-vegetated site (if logistically possible), the seagrass site can be done another day.

3. Lay out three x 50m tape measures perpendicular (*not* parallel) to the shoreline and 25m apart (see figure 2).
The three tapes should be parallel to each other.
Use pegs, sticks or weights (dive weights, stones, etc.) to hold the tapes in place. Pegs or sticks could for example be made of rebar (metal bars for construction) that is cut into smaller pieces.
If you only have one measuring tape, do one transect at the time, but place all pegs for the three transects from the start, or keep the pegs in the sediment from the first transect to facilitate placement of the remaining transects.

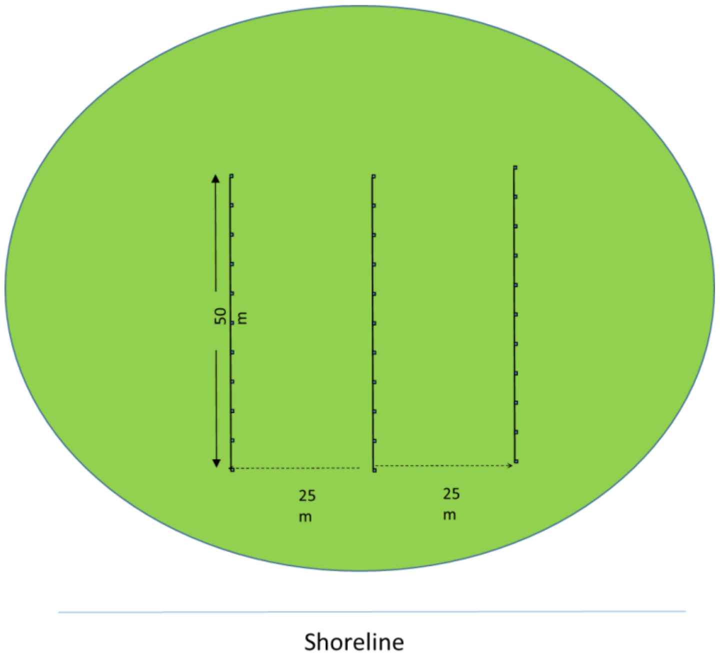

Figure 2. Transect and quadrat layout for SeagrassWatch sampling. The transects are placed perpendicular (not parallel) to the shore.

4. If you use the ODK collect app, please follow the instructions, starting with the first transect. The app will guide you through the sampling.
There are one ODK collect form for the large invertebrates and one form for the ecological survey of seagrass, un-vegetated and small invertebrates.

5. Mark the position of the start and end of each transect using the GPS in your Android device, or with your handheld GPS. Please note it may take >60 seconds for the GPS to find enough satellites. You should aim for at least an accuracy of 10 meters on the GPS. The GPS point and time should be taken once you start working with your transect.

6. When the transects are in place, first, survey large invertebrates along the transect. Identify and count all large living invertebrates (>5cm) found within 1 meter to each side of the transect. This large invertebrate survey is done for all three transects in both seagrass and un-vegetated sites. We suggest that you first survey the large invertebrates along the transects and then conduct the ecological survey, before moving on to the next transect. Walk along each transect on the left side of the tape (when starting from the shore walking towards deeper waters). It is very important, as you will survey seagrass/un-vegetated and small invertebrates on the right-hand side of the tape with 0.5x0.5m quadrats.
If the water movement results in that sediments stir into the quadrats, plan placing the quadrats on the left-hand side instead, and walk on the right-hand side. Please find a detailed protocol below.

7. Second, survey seagrass/un-vegetated and associated small-invertebrates (>1cm) with quadrats. Start at the beginning of the transect at 0 m, place the 0.5x0.5m quadrat to the right hand side of the tape (see figure 3). Assess all parameters (e.g. seagrasses, macrophytes and sediment type) within the 0.5x0.5m quadrat every 5 m (at 0, 5, 10, 15 m etc.). If there is seagrass in the quadrat you should also assess total % cover, seagrass species composition, canopy height and epiphytes. When you finish the transect you should have data from 11 quadrats along the transect line. The last quadrat is placed between 50-50.5m (i.e. placed outside the transect line of 50 meters).

If there is no seagrasses, macrophytes and epiphytes indicate with 0 (zero). In the un-vegetated (reference) site this sampling will be very quick if you have no/very little vegetation.

8. While you conduct the seagrass/un-vegetated survey as you work your way along the transect, also survey smaller invertebrates (larger than 1cm) within the same sampling quadrate (0.5x0.5 m frame) at 4 pre-defined points along each transect; at 0m, 15m, 35 m and 50m.

9. When you finished the both surveys, large invertebrate and ecological survey of small inverts and seagrass/unvegetated, along one transect please make sure you have filled in all the data and save (if you are using the app). Then continue with the next transect.

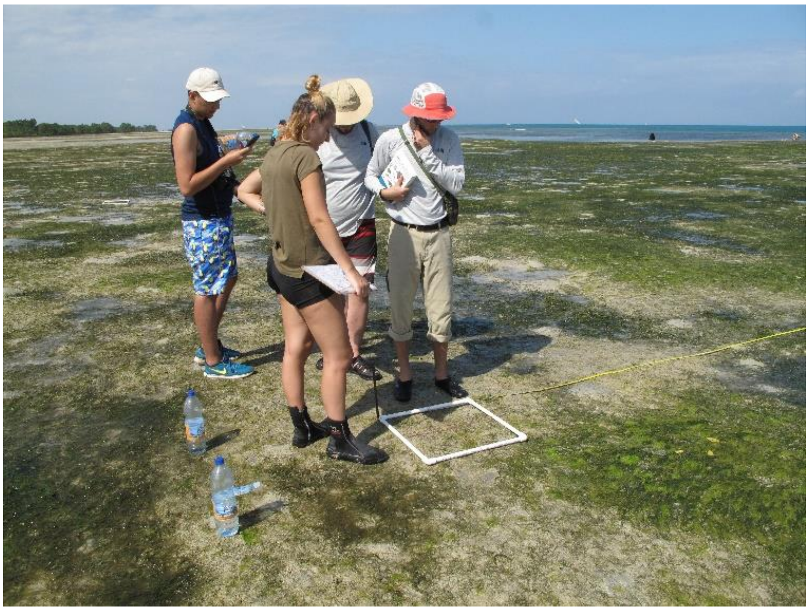

Figure 3. Quadrats should be placed flush to the right hand side of the tape measure whilst the observer (s) stands to the left. The start of the transect is closest to shore. Looking towards the ocean, you will be able to identify the right and left side of the transect.

Detailed description of large invertebrate sampling

At the un-vegetated site and the seagrass site you should survey the large invertebrates over a larger sampling area (belt transect 50*2meters). First step is to lay out the 3 transects in exactly the same format as for SeagrassWatch (figure 2), i.e. perpendicular to the shore and 25 m apart.
The assessment of large invertebrates is done along the whole transect. It is advisable to do this survey right after the transects have been placed, thus before you survey the seagrass/unvegetated and the small invertebrates.

Along each of the three transects at both sites you will need to identify (ideally to species-level, and if not possible, to genus or family) and count all large invertebrates (here: live animals >5 cm length or diameter) that occur within a band of 1m to each side of the tape measure (i.e. a belt transect of 2x50 m = 100m^2). If needed, use the 1.0 m long stick (and measure from the transect tape or use your quadrate – see instruction video) to decide whether individual animals are within our outside of the 2m wide belt transect.
Please record both taxa and species name where possible. The list of taxa presented in table 1 is available as first level choice in the app. Record the abundance (number of individuals) of each species.
Typical taxa that you are likely to find will include large echinoderms (sea urchins, sea stars, sea cucumbers, etc.), large bivalves (e.g. fan mussels or giant clams), gastropods (e.g. cowries, helmet shells), squid, etc.
Make sure not to count empty (dead) shells.

Table 1. List of taxa available in the app.
This is the minimum level of identification.
It is very important that you familiarize yourself with the taxa in the table.

**Taxa (the minimum level when identifying species in the catch)**
Bivalves (Bivalvia)
Snails & sea slugs (Gastropoda)
Squids, cuttlefish and octopi (Cephalopoda)
Prawns & shrimps (Caridea & Dendrobranchiata)
Lobsters , crayfish & similar (Astacidea)
Crabs (Brachyura)
Sea urchins (Echinoidea)
Sea cucumbers (Holothuroidea)
Starfish (Asteroidea)
Brittlestars (Ophiuroidea)
Worm-like organisms excluding fish
Fish, bony fish (Osteichthyes)
Sharks & rays and other cartilaginous fish (Elasmobranchii)
Turtles & Sea snakes (Reptilia)
Marine mammals (Mammalia)
Sponges (Porifera)
Cnidarians – eg. Coral and jelly fish (Cnidaria)
Other Crustaceans (not already mentioned)
Other Mollusca – eg. Chitons, tusk shells (not gastropods)
Other Echinodermata – eg. featherstars
Other

Identification of these animals can be difficult, therefore also the possibility to only record taxa (table 1), but the more information we collect about the invertebrates the better it is. Depending on the location and the local regulatory rules it might be easier to collect the animals in sample jars and preserve them for identification in the laboratory. It may also be possible to take good photos and notes in the field, noting particular details later used for identification. Naturally, it is crucial to note which transect (T1, T2 or T3) and site each collected specimen belongs to.

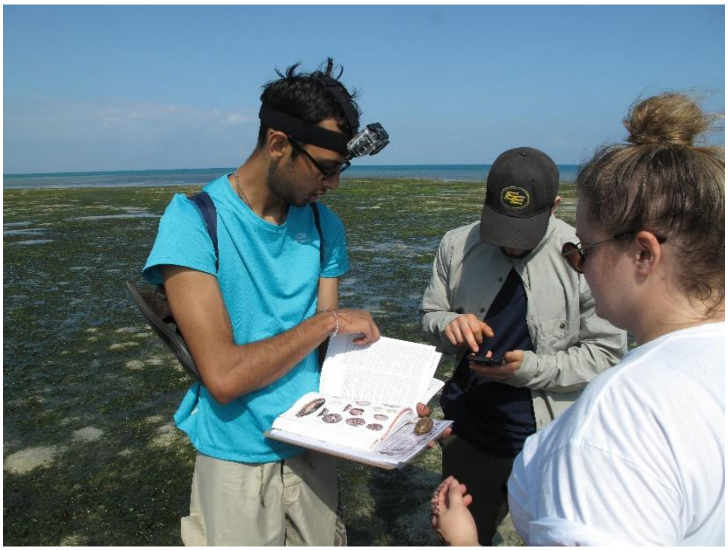

Figure 4. Identifying invertebrates to lowest taxonomical level possible. If you are unsure of the species, it is for example better to record family instead of species name. Remember there are many more species than present in identification guides and books.

Detailed description of small invertebrate sampling

Along each of the three transects in both the un-vegetated site and the seagrass site you will need to sample 4 of the 11 quadrats (at 0, 15, 35 and 50 m) for small invertebrates, using a smaller 0.5x 0.5m quadrat covering 0-0.25m; 15-15.25m; 35-35.25m & 50-50.25m along the transect. This sampling is conducted while you are doing the seagrass and unvegetated quadrates.

In each of the 0.5x0.5m quadrats you will need to identify (ideally to species-level) and count all living invertebrates. Only include invertebrates larger than 1 cm in body length/width (invertebrates smaller than 1 cm should not be counted or identified). Record both taxa and species name if possible. See table one for list of taxa - minimum level for identification. Invertebrate taxa you may expect to find in these quadrats are different types of bivalves, gastropods, crustaceans (for example, hermit crabs), echinoderms, sea cucumbers, rag worms, corals and sponges etc. It is important that you include all invertebrates that you find, and not only the species (taxa) that are gleaned.

Start by identifying, counting and removing all invertebrates visible within the quadrat. Note that individual invertebrates could be found between seagrass shoots on the sediment surface, or directly on the seagrass shoots. Then, using your fingers (preferably wearing protective gloves to avoid cuts and stings), search through the upper 2 cm sediment layer and dig out all encountered invertebrates. Make sure to only identify and count *live* organisms (for example, do not count empty bivalve or gastropod shells). If it is difficult to dig out the invertebrates with your hands, use a knife or a sharp stick to dig.

NOTE: Make sure you inform yourself about hazardous species in your area, and avoid any contact with those species. Example of species that can be dangerous to humans are certain types of Conus, a gastropod that looks like a cone, and sea urchins.

Identification of these invertebrates may be difficult. Depending on the location and the local regulatory rules it might be easier to collect the animals in sample jars/containers (one jar per quadrat) and preserve them for identification (e.g. in 70% ethanol) in the laboratory. It is always good to take pictures of the animals in the field, but remember to note (preferably included in the picture) the ID of the quadrat they are found in. Quadrats IDs are T1:0 (transect 1 at 0 meters), T1:15, T1:35, T1:50, T2:0, T2:15 etc.

Different invertebrate classes (e.g. Bivalvia, Gastropoda) have different characteristics that are needed for invertebrate identification; therefore, it is important to record (or take photos of) these characteristics. For example, for bivalves the characteristics of hinge and umbo might be needed for identification. Please see the IPSN guide for species ID to learn more about what characteristics might be important for different animal groups (guide under development – in the meantime look in any invertebrate identification guide).

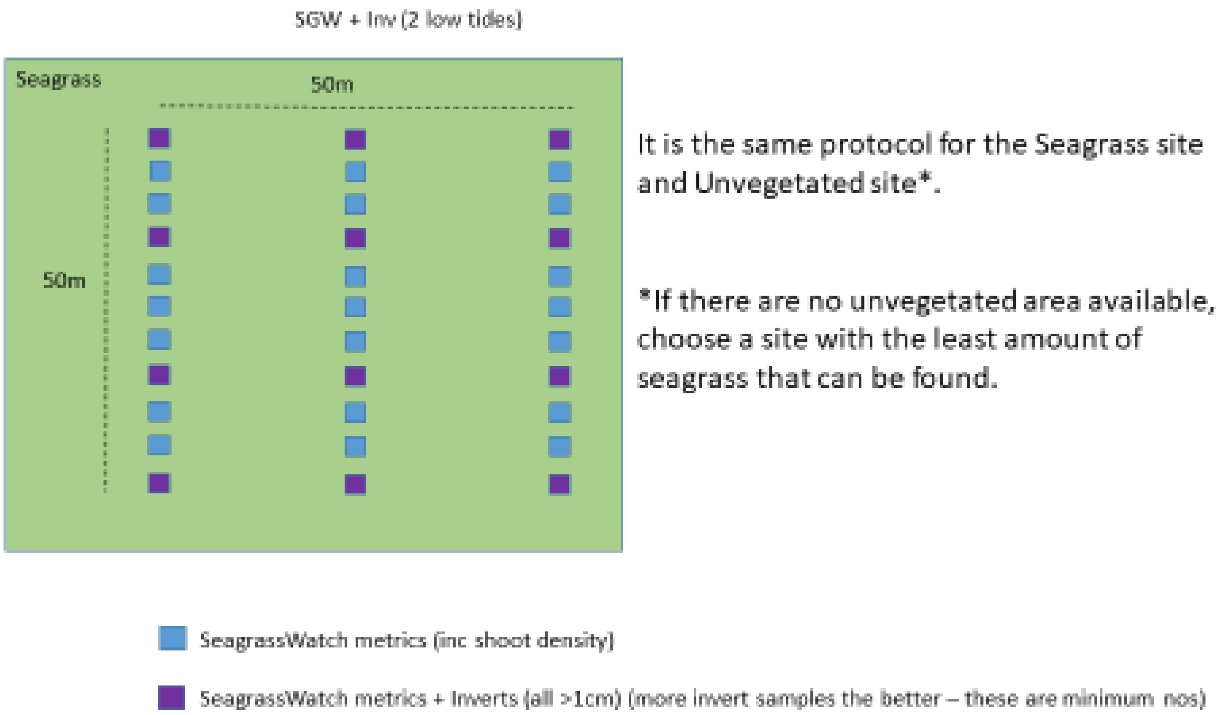

Figure 5. Configuration of the seagrass and invertebrate sampling within quadrats at the seagrass and at the un-vegetated (bare sand or mud) sites.

Detailed description of the un-vegetated and seagrass survey

In the un-vegetated site, please record seagrass (if present record all seagrass parametrics), algae (if present), sediment type, water level and any other relevant features, along with invertebrates. In the unvegetated site follow all the steps in the seagrass guide, but indicate with 0 (zero), if seagrass is not present in the quadrate.

In the seagrass site, you will sample several standard seagrass metrics within a 0.5x0.5m quadrat, that is laid out at 11 points (starting at 0, and then every 5 m) along each of the 3 transects. This results in a total of 33 seagrass quadrats overall in the site.

Within each 0.5x0.5m quadrat, the observer will assess the metrics as outlined below.

First, place the seagrass quadrat with one side along the transect line, so that the bottom-right corner starts at “0m”.

<u>Seagrass percent cover:</u> Looking down on the quadrat from above, estimate the total percentage (0-100%) of the seabed within the quadrat that is covered by seagrass (pooling all seagrass species), to the closest 1%.

It is important that you use the percent cover photo standards (calibration sheets) here as a guide.
When estimating cover, make sure to exclude the footprint/shadow provided by the seagrass shoots.
Make sure not to stir up sediment that will hinder your view of the seagrass in the quadrat or other areas of the meadow.

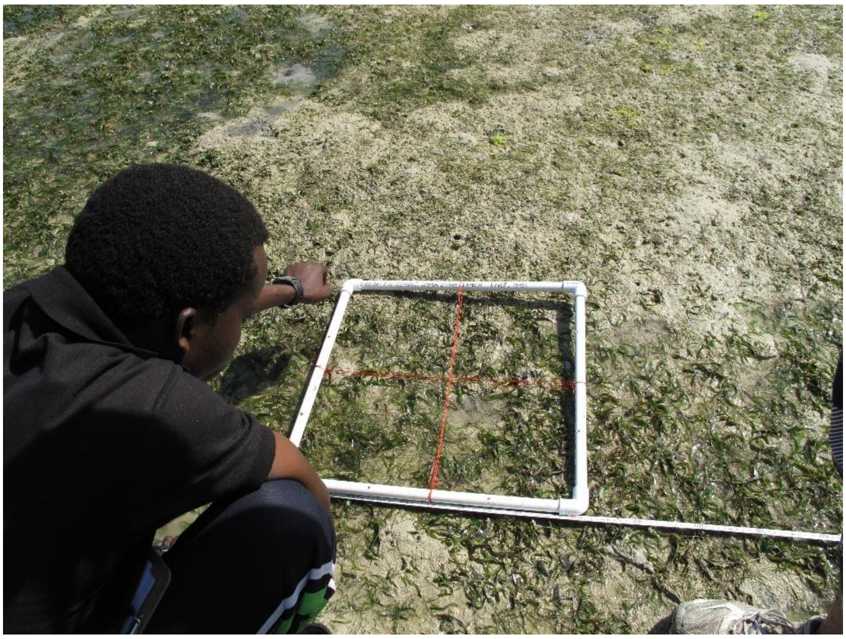

Figure 6. Estimating seagrass cover. It is optional to divide the quadrat into 4 compartments, this can sometimes facilitate during estimation of seagrass and algal cover.

Algae percent cover: Estimate % cover of algae in the quadrat using the “Algal percentage cover photo guide”. Algae are here defined as seaweeds that are not attached to seagrass but may cover or overlie the seagrass blades.
Macroalgae percentage cover is independent of seagrass cover; for example, you can have 100% seagrass cover and 100% macroalgae cover (drift) in the same quadrat.

Epiphyte percent cover: Epiphytes are algae attached to seagrass blades and can either give the blade a furry appearance (filamentous algae), or occur as a crust-like, greyish sheath (coralline algae).
First estimate how much of the blade surface is covered by epiphytes, and then what proportion of the blades in the quadrate are covered (e.g., if 20% of the blades are each 50% covered by epiphytes, then quadrat epiphyte cover is 10%).
Do not include epifauna with epiphytes. Epifauna are sessile animals (e.g. worms, sponges, etc.) attached to seagrass blades – please record % cover of epifauna in the comments or an unused/blank column – do not add to epiphyte cover.
Use the epiphyte matrix to help you in the field. (DOWNLOAD: Epiphyte_matrix.pdf)

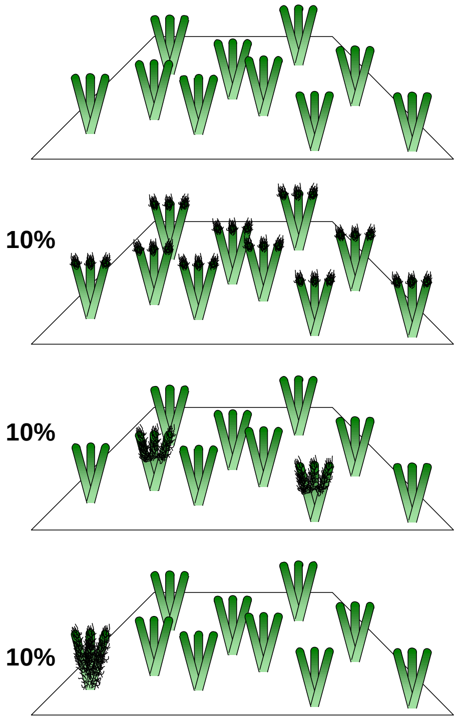

Figure 6. Example illustration of the estimation of epiphytes % cover (Figure source: SeagrassWatch)

<u>*Seagrass species composition:*</u> Identify the species of seagrass growing within the quadrat and determine the percent contribution of each species to the total cover.
Example: total seagrass cover in a quadrat in a mixed *Thalassia hemprichii*/*Thalassodendron ciliatum* meadow is 73%. Meanwhile , 90% of that cover is made up of *Thalassia*, and 10 % of *Thalassodendron*.
Use seagrass species identification keys provided, and use more than one feature to identify the species. Note that total composition must equal 100%.

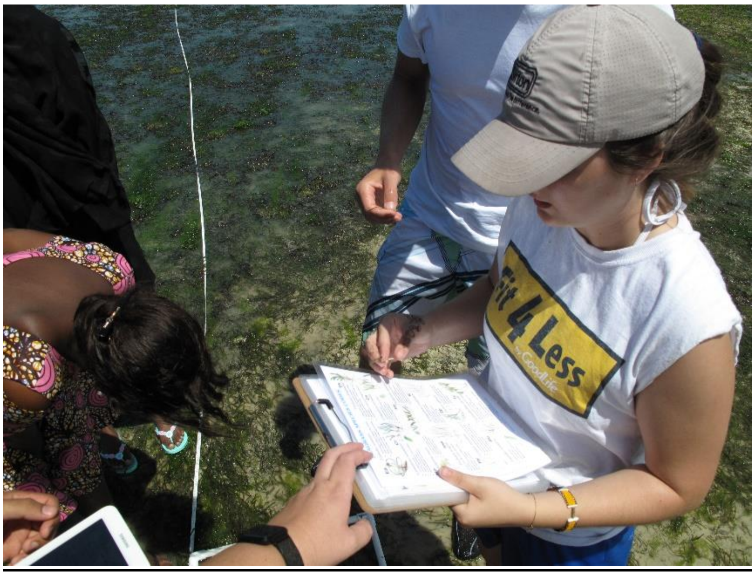

Figure 7. Identifying seagrass species guided by SeagrassWatch seagrass species identification keys.

Canopy height: Measure seagrass canopy height (to the closest 0.1 cm) of the dominant strap-leaved species, while ignoring the tallest 20% of leaves, by gently grabbing the leaves and extending them upwards.
Measure from the sediment surface to the leaf tip of at least three (3) mature leaf blades (strap leaved species).
Write the three (3) measures in the Android app or on the data sheet.

Sediment: To assess the sediment coarseness, dig your fingers into the top centimetre of the substrate and feel the texture. Describe the sediment, by noting the grain size in order of dominance (e.g., Sand, Fine sand, Fine sand/Mud).

Water depth:
If more than 50% of the quadrat is covered by water, measure and record the water depth in cm. No water should be recorded as 0.

Other features:
Note and count any other features (e.g. evidence of turtle feeding, 'burning' (browning) of leaves) which may be of interest. In a usual SeagrassWatch survey the observer would at this point assess the macrofauna, but in this instance you do not need to do this as you will be doing this using separate protocols (see below).

Voucher seagrass specimen collection (if required for identification)
Voucher specimens are only required if: establishing monitoring in a new region; a new seagrass species is present; or if you are unsure of the seagrass identification.
Seagrass samples should be placed inside a labelled plastic bag with seawater and a waterproof label.
Select a representative specimen of the species and ensure that you have all the plant part including the rhizomes and roots.
Collect plants with fruits and flowers structures if possible.
Take photos of the sample in the field if possible.

Once you have completed the surveys in the un-vegetated and seagrass sites, you should have the following sets of data from both sites:

A. From each of the 33 (11 quadrats x 3 transects) large (0.5x0.5m) quadrates:

- % total seagrass cover (0-100%)
- Scientific names of all seagrass species present, and their relative (%) contribution to total cover
- Macroalgae cover (0-100%)
- % epiphyte cover (0-100%)
- Seagrass shoot density (# of shoots growing within the quadrat)
- 3 estimates of seagrass canopy height (closest 1 cm)
- Estimate of sediment composition (e.g. sand, mud, course sand)

If your unvegetated site has no seagrass, macroalgae or epiphytes at all (the ideal situation for this site), you can very quickly note this.

B. From each of the 12 (4 quadrats x 3 transects) small (0.25x0.25m) quadrats:

- Number of invertebrates (larger than 1 cm) per quadrat split up between each taxa (with scientific name in latin). Include the invertebrates found in the top layer of the sediment, among the shoots or on the shoots ,

C. From each of the three 50x2 m belt transects along each transect line:

- Number of large epifauna individuals per belt transect, split up between each taxa (with scientific name in latin)

Definitions:

Location: Location is the area you and your team have chosen as the place where you want to conduct IPSN related research. The location includes the whole seascape and the adjacent village or town.
Site: Site is the area in the intertidal zone or water where you decide to collect your data.
Seagrass site: A site that is dominated by seagrass and preferably larger than 50x50 meters. The area can be patchy but should be dominated by seagrass.
Un-vegetated site: A site with bare sediment, without seagrass or other vegetation. This area should be as similar to the seagrass site as possible but without the seagrass. Preferably the same water depth, the same distance from shore, the same type of habitat as the seagrass in the seagrass site is growing is.

References:

Information contained in this IPSN research protocol about SeagrassWatch was taken from the SeagrassWatch manual:

McKenzie, L.J. & Campbell, S.J. (2002) Seagrass-Watch: Manual for Community (citizen) Monitoring of Seagrass Habitat. Western Pacific Edition (QFS, NFC, Cairns) 43pp

Please refer to this guide as:

Eklöf, Nordlund & Unsworth. IPSN research protocol: Ecological surveys of seagrasses and invertebrates. October 2018.
